# SARS-CoV-2 propagation to the TPH2-positive neurons in the ventral tegmental area induces cell death via GSK3β-dependent accumulation of phosphorylated tau

**DOI:** 10.1371/journal.pone.0312834

**Published:** 2024-10-30

**Authors:** Motoki Imai, Fumitaka Kawakami, Takayuki Uematsu, Toshihide Matsumoto, Rei Kawashima, Yoshifumi Kurosaki, Shun Tamaki, Shotaro Maehana, Takafumi Ichikawa, Hideaki Hanaki, Hidero Kitazato, Makoto Kubo

**Affiliations:** 1 Department of Molecular Diagnostics, School of Allied Health Sciences, Kitasato University, Sagamihara, Kanagawa, Japan; 2 Department of Applied Tumor Pathology, Graduate School of Medical Sciences, Kitasato University, Sagamihara, Kanagawa, Japan; 3 Regenerative Medicine and Cell Design Research Facility, School of Allied Health Sciences, Kitasato University, Sagamihara, Kanagawa, Japan; 4 Department of Regulation Biochemistry, Graduate School of Medical Sciences, Kitasato University, Sagamihara, Kanagawa, Japan; 5 Department of Health Administration, School of Allied Health Sciences, Kitasato University, Sagamihara, Kanagawa, Japan; 6 Biomedical Laboratory, Division of Biomedical Research, Kitasato University Medical Center, Kitamoto, Saitama, Japan; 7 Department of Pathology, School of Allied Health Sciences, Kitasato University, Sagamihara, Kanagawa, Japan; 8 Department of Biochemistry, School of Allied Health Sciences, Kitasato University, Sagamihara, Kanagawa, Japan; 9 Department of Clinical Chemistry, School of Allied Health Sciences, Kitasato University, Sagamihara, Kanagawa, Japan; 10 Department of Environmental Microbiology, Kitasato University Graduate School of Medical Sciences, Sagamihara, Kanagawa, Japan; 11 Infection Control Research Center, Ōmura Satoshi Memorial Institute, Kitasato University, Minato-Ku, Tokyo, Japan; Max Delbruck Centrum fur Molekulare Medizin Berlin Buch, GERMANY

## Abstract

COVID-19, an infectious disease caused by SARS-CoV-2, was declared a pandemic by the WHO in 2020. Psychiatric symptoms including sleep disturbance, memory impairment, and depression are associated with SARS-CoV-2 infection. These symptoms are causes long-term mental and physical distress in recovering patients; however, the underlying mechanism is unclear. In this study, we determined the effects of SARS-CoV-2 infection on brain tissue using k18hACE2 mice. Using brain tissue from 18hACE2 mice infected with SARS-CoV-2 through intranasal administration, SARS-CoV-2 spike protein and RNA were analyzed by immunohistochemical staining and in-situ hybridization. Immunohistochemical analysis revealed that Tryptophan hydroxylase 2 (TPH2)-positive cells and SARS-CoV-2 spike protein were co-localized in the ventral tegmental area of SARS-CoV-2-infected mice. We observed decreased TPH2 expression and increased accumulation of phosphorylated tau protein and Phospho-Histone H2A.X (γH2AX) expression in the ventral tegmental region. In addition, activation of glycogen synthase kinase 3β (GSK3β) was induced by SARS-CoV-2 infection. Overall, our results suggest that SARS-CoV-2 infection of TPH2-positive cells in the ventral tegmental area induces neuronal cell death through increased accumulation of phosphorylated tau. Attenuation of the GSK3β pathway and decreased serotonin synthesis through suppression of TPH2 expression may contribute to the development of neurological symptoms.

## Introduction

COVID-19 is an infectious disease caused by SARS-CoV-2, which was declared a pandemic by the WHO on March 11, 2020. To date, more than 774 million people have been infected worldwide and more than 7 million have died (as of World Health Organization 2024.1.7). Symptoms of SARS-CoV-2 infection are characterized by pneumonia and respiratory effects due to upper respiratory tract infection; however, some SARS-CoV-2-infected patients have neurological symptoms characterized by headache, dizziness, sleep disturbances, psychosis, anxiety, and depression, even after recovery from the acute phase of infection [1]. Thus, there are SARS-CoV-2-infected patients suffering from sequelae after SARS-CoV-2 infection without complete recovery of symptoms [2].

SARS-CoV-2 infection may affect the brain in multiple ways [3–5] and it may induce neuronal dysfunction and death in the brain [6] and such nerve damage may result in neuropsychiatric dysfunction. Serotonin is a neurotransmitter closely associated with depression, Alzheimer’s disease, and other neurological disorders [7, 8]. Serum serotonin levels have been shown to be decreased in SARS-CoV-2-infected patients, suggesting abnormal serotonin metabolism [9]. A deficiency in serotonin levels may be the cause of olfactory impairment. Tryptophan hydroxylase 2 (TPH2) is the rate-limiting enzyme that synthesizes serotonin from tryptophan [10]. TPH2 expression is closely associated with neuropsychiatric symptoms, such as anxiety and depressive-like behavior, as decreased TPH2 expression induces reduced serotonin synthesis [11].

Approximately 30% of SARS-CoV-2-infected patients have some neuropsychological symptoms, such as fatigue, brain fog, memory impairment, attention disorders, and sleep disturbances three months after SARS-CoV-2 infection [12]. Among these neuropsychiatric symptoms, the prevalence of psychiatric symptoms, primarily sleep disturbances, anxiety, and depression, remain high, even 12 months after infection. Thus, patients infected with SARS-CoV-2 that experience neurological symptoms are unlikely to return to work or lead a normal daily life [13]. Neurological symptoms that occur after SARS-CoV-2 infection are a major obstacle to quality of life [14] and prevent full social reintegration for many SARS-CoV-2-infected individuals [15].

In this study, we used the human transgenic 18hACE2 mice model has been used widely to test the efficacy SARS-CoV-2 infection. In addition, Hassler, Luise et al. reported increased leukocytosis and/or endothelial hypertrophy in the striatum, cerebral cortex, and hypothalamus due to SARS-CoV-2 infection [16]. The SARS-CoV-2 strain, hCoV-19/Japan/TY/WK-521 strain (WK-521, Pango Lineage A), which was isolated from patients with COVID-19 [17], was using this study.

In the present study, we determined the effects of SARS-CoV-2 infection on the expression of TPH2, the rate-limiting enzyme of serotonin, which is closely associated with neuropsychiatric symptoms, such as depression, as well as its effects on nerve damage. Furthermore, we examined one aspect of the pathogenesis of neuropsychiatric symptoms by determining the effects of SARS-CoV-2 infection on the central nervous system.

## Materials and methods

### Animal experiments

#### Mice

k18hACE2 mice have been previously described [18]. Heterozygous k18hACE2 mice were purchased from the Jackson Laboratory (Bar Harbor, ME). The animals were cared under specific pathogen-free conditions and handled in according to the protocol approved by the President of Kitasato University after review by the Institutional Animal Care and Use Committee (approval number: 2020–8, 2021–9, 2022–9), and Genetic Modification Experiment Safety Committee (Approval number 4557, 5205). The health of the mice was monitored at least once a day and no accidental deaths were observed. All staff and researchers at the animal facilities received the necessary training to carry out animal experiments.

#### SARS-CoV-2

The SARS-CoV-2 strain, hCoV-19/Japan/TY/WK-521 strain (WK-521, Pango Lineage A), which was isolated from patients with COVID-19 [17], was provided by The National Institute of Infectious Diseases (Tokyo, Japan).

#### SARS-CoV-2 infection in mice

Heterozygous k18hACE2 mice (8–12 weeks old) were anesthetized by intraperitoneal administration with medetomidine hydrochloride (0.75 mg/kg), midazolam (4 mg/kg), and butorphanol tartrate (5 mg/kg) and infected with 5 × 10^4^ PFUs (Plaque Forming Units) of SARS-CoV-2 (hCoV-19/Japan/TY/WK-521) by intranasal administration. The mice were inoculated with D-MEM (Fujifilm Wako Pure Chemical, Osaka, Japan) supplemented with 2% fetal bovine serum (Life Technologies, Carlsbad, CA) and antibiotics (100 IU/mL penicillin and 100 μg/mL streptomycin; Fujifilm Wako Pure Chemical). The mice were euthanized by intraperitoneal administration of sodium pentobarbital (150 mg/kg) on day 7 post-infection and brain tissue was collected. The humane endpoint was to be applied when the cumulative weight loss during the 2–3 day observation period exceeded 20% and the individual was excessively debilitated due to an excessive inflammatory response. For affected individuals, it was planned that the experiment would be terminated and euthanasia by overdose of sodium pentobarbital would be promptly chosen, but no applicable cases occurred in the present study. The experiments were performed in an animal biosafety level 3 (ABSL3) facility of Kitasato Medical center.

#### Ethical approval statement

The animal experimental protocols were approved by the Kitasato University Animal Care and Use Committee (Approval No. 2020–8, 2021–9, 2022–9) and the Kitasato University Genetic Modification Experiment Safety Committee (Approval No. 4557, 5205) in agreement with internationally accepted standards and followed the recommendations in the ARRIVE guidelines. All experiments were performed in accordance with relevant guidelines and regulations.

### Immunohistochemistry

Brain tissues obtained from SARS-CoV-2 infected mice were fixed in 10% formalin for 48 h, paraffin-embedded at Geno Staff Co. Ltd., and sectioned at 3 μm. Sections were deparaffinized, rehydrated, and microwaved or autoclaved (121°C, 10 min) in 0.01 M sodium citrate buffer (pH 6.0) for antigen retrieval. Then, 3% hydrogen peroxide was used to inhibit endogenous peroxidase activity. The brain sections were blocked with protein block serum-free ready-to-use (Dako) for 1 h at room temperature. Primary antibody (anti-TPH-2: 101962, Gene Tex, SARS-CoV-2 spike: 40589-T62, Sino Biological, Inc.) was diluted 1:500 in antibody diluent with background reducing components (Dako, CA, USA) and incubated overnight at 4°C. After washing with PBS three times for 5 min each, secondary antibodies (Dako, CA, USA) were incubated with the tissue sections for 1 h at RT. The ImmPACT^®^ DAB substrate kit (VECTOR) was used for signal development.

### In-situ hybridization

SARS-CoV-2 RNA expression was analyzed using the RNAscope assay (Advanced Cell Diagnostics, Hayward, CA, USA). The brain sections were deparaffinized and incubated with target retrieval solution for 15 min at 98ºC and then treated with Proteinase Plus for 30 min at 40ºC. Hybridization was used the following probes: V-nCov2019-S-sense (RNAscpoe^®^ Probe: 845701) and negative control probe (RNAscpoe^®^ Probe: 848561) for 2 h at 40ºC. The signals were visualized by incubating with DAB and the nuclei were contrast stained with hematoxylin.

### Immunofluorescent staining

The brain sections were deparaffinized, rehydrated, and microwaved in 0.01 M sodium citrate buffer (pH 6.0) for antigen retrieval. The sections were blocked with protein block serum-free ready-to-use (Dako, CA, USA) for 1 h at room temperature. Primary antibody (anti-TPH-2: 91108, SIGMA, SARS-CoV-2 spike: 40589-T62, Sino Biological Inc.) was added at a 1:500 dilution in antibody diluent with background reducing components (Dako, CA, USA) and incubated overnight at 4°C. After washing with PBS three times for 5 min each, secondary fluorescence-labeled antibodies (anti-rabbit IgG Alexa fluor 488: 4412S, anti-mouse IgG Alexa fluor 594: 8890S, cell signaling technology, USA) were added at a 1:500 dilution and DAPI (D532, Dojindo) at a 1:1000 dilution in antibody diluent with background reducing components (Dako, CA, USA) and incubated for 1 h at RT. Tissue sections were washed with PBS three times for 5 min and an additional two times with dH2O and mounted with fluorescence mounting medium (Dako, CA, USA).

### TUNEL staining

TUNEL staining was performed using the TUNEL assay kit (#25879, CST) according to the manufacturer’s instruction, followed by staining with DAPI (D532, Dojindo). Finally, the brain sections were washed by distilled water and mounted for visualization and obtain fluorescent images. The number of TUNEL positive cells were counted using ImageJ.

### Western blot analysis

Brain tissue samples collected from SARS-CoV-2-infected mice were washed thoroughly with PBS prior to lysate preparation. The samples were solubilized in RIPA buffer (Fujifilm Wako Pure Chemical Corporation) supplemented with HALT^®^ protease and phosphatase inhibitor cocktail (Thermo Fisher Scientific). Western blot was performed as described previously [19]. The primary antibodies included SARS-CoV-2 spike (40589-T62, Sino Biological Inc.), Tryptophan hydroxylase 2 (TPH2) (101962; Gene Tex), γ-H2AX (S139, 9718S; Cell signaling technology), P-Tau (S404, ab92676; abcam), T-Tau (ab109392; abcam), P-GSK-3β (S9, 5558S, Cell signaling technology), T-GSK-3β (22104-1-AP; Proteintech), P-AKT (S473, 4060S; Cell signaling technology), T-AKT (9272S; Cell signaling technology), Caspase-3 (14220S; Cell signaling technology) at dilutions ranging from 1:1000–1:2000. The secondary antibodies included HRP donkey anti-mouse IgG (H+L) antibody (Jackson Immuno Research), HRP donkey anti-rabbit IgG (H+L) antibody (Biolegend), and HRP donkey anti-goat IgG antibody (Santa Cruz Biotechnology) diluted 1:5,000.

### Statistical analysis

The data were analyzed using GraphPad Prism 8 (GraphPad Software). All experimental data were presented as means ± standard deviation (SD). P values were calculated using a Student’s t-test and significance was set at P <0.05.

## Results

### SARS-CoV-2 spreads transneuronally in the brain of k18hACE2 mice

**Fig 1:**
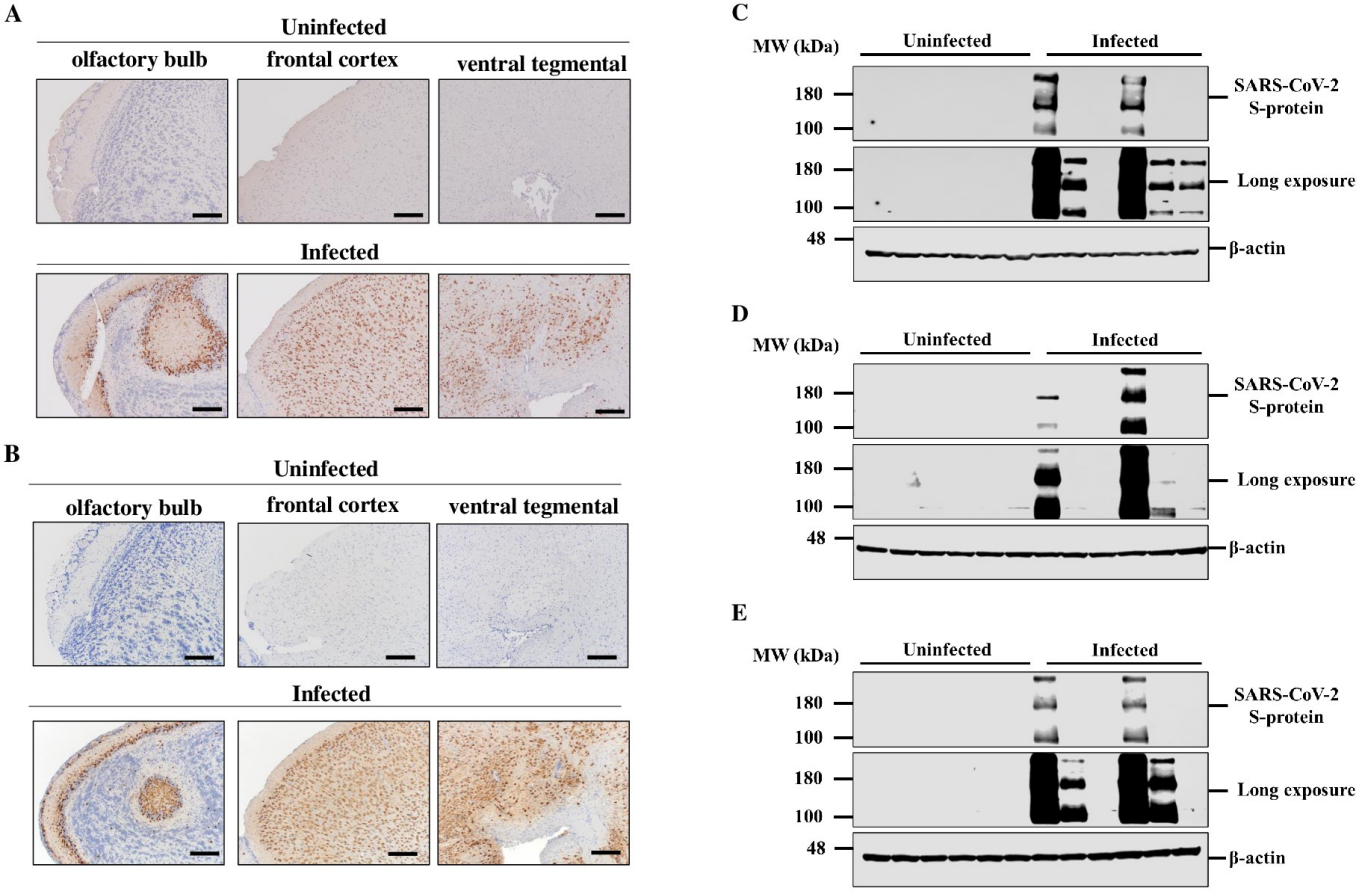
SARS-CoV-2 infected K18-hACE2-Tg-mouse brain. Immunohistochemistry staining of (A) SARS-CoV-2 spike protein, (B) SARS-CoV-2 RNA in-situ hybridization of brain sections from k18hACE2 mice uninfected or infected with 5 × 10^4^ PFU 7 days after administration. Bar is 200 μm. Western blot analysis of SARS-CoV-2 spike protein in the (C) frontal cortex, (D) olfactory blub, and (E) VTA from brain tissue lysates of k18hACE2 mice in uninfected or infected with 5×10^4^ PFU 7 days after administration. The data are representative of 6 mice per group.

We determined whether intranasally administered SARS-CoV-2 infects the brain of k18hACE2 mice by immunohistochemical staining of SARS-CoV-2 spike protein and in-situ hybridization of SARS-CoV-2 mRNA. Immunohistochemical staining revealed SARS-CoV-2 spike protein-positive cells in the cerebral cortex, olfactory bulb, and VTA of mice administered SARS-CoV-2 (Fig 1A). SARS-CoV-2 nucleic acids were also detected by in-situ hybridization in the cerebral cortex, olfactory bulb, and VTA, which confirmed that SARS-Cov2 infects the brain of k18hACE2 mice (Fig 1B). Furthermore, SARS-CoV-2 was detected by western blot analysis in brain tissue lysates from SARS-CoV-2-infected mice. The results indicated that SARS-CoV-2 infected the cerebral cortex (Fig 1C), olfactory bulb (Fig 1D), and even the VTA (Fig 1E), which is consistent with the results of immunohistochemical staining and in-situ hybridization.

### SARS-CoV-2 infects TPH2-positive cells in the ventral tegmental area

**Fig 2:**
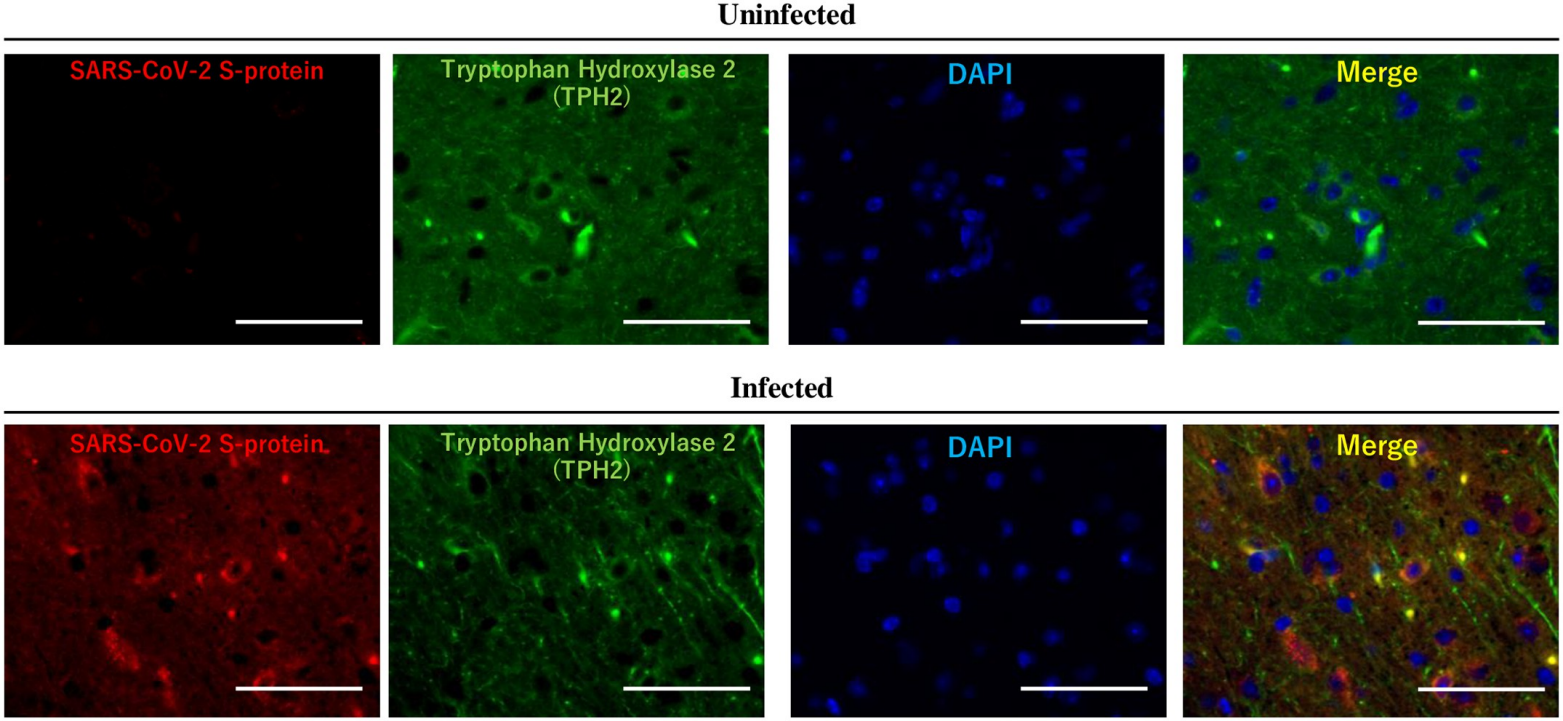
SARS-CoV-2 infected Tryptophan hydroxylase 2 (TPH2)-positive cells in the ventral tegmental area. Immunohistochemistry staining analysis of SARS-CoV-2 spike protein: red, TPH2: green, and DAPI: blue in the VTA of brain sections from k18hACE2 mice uninfected or infected with 5×10^4^ PFU 7 days after administration. Bar is 50 μm.

**Fig 3:**
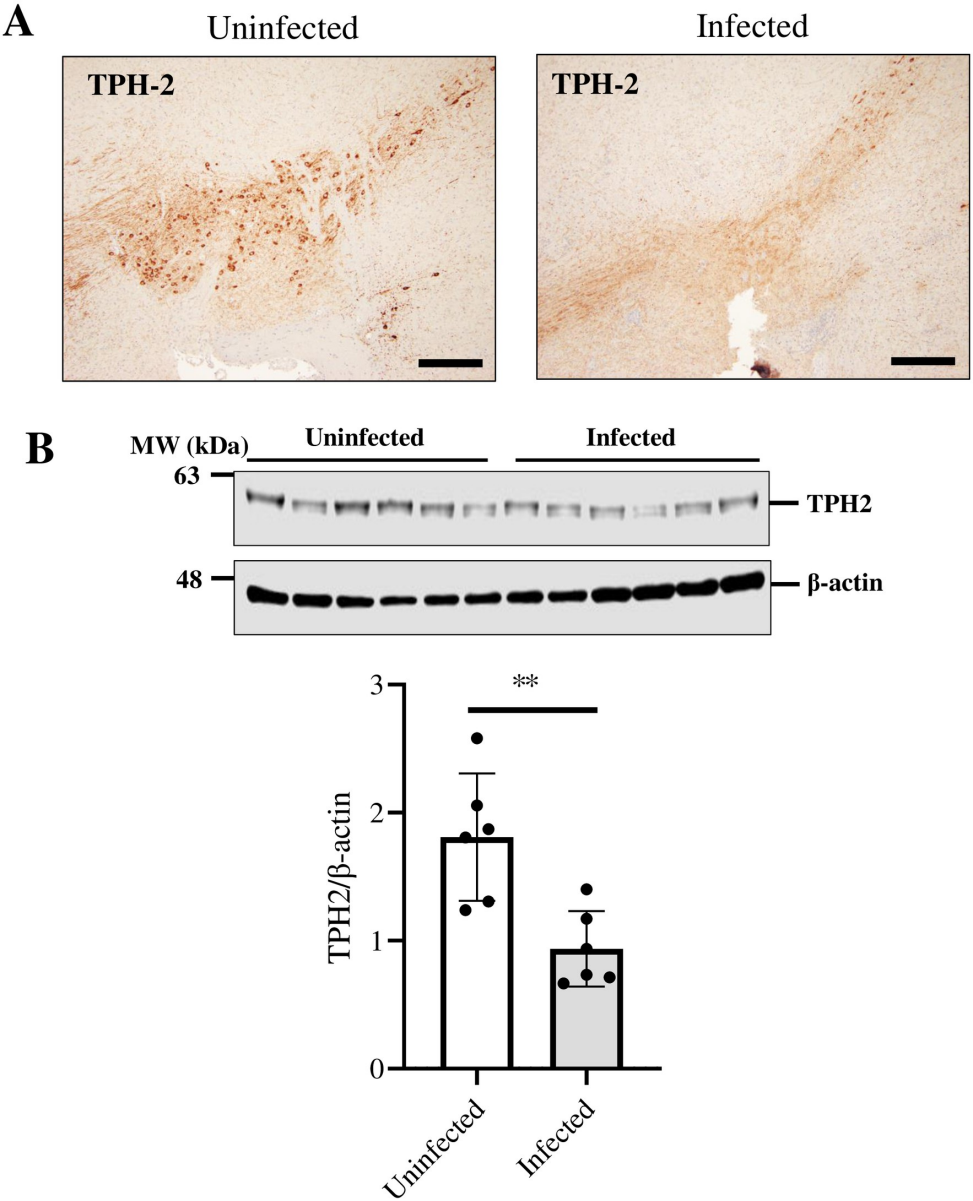
The effect of SARS-CoV-2 infection on Tryptophan hydroxylase 2 (TPH2) expression in the k18hACE2 mice ventral tegmental area. (A) Immunohistochemistry staining analysis of TPH2 in the Vt area of the brain section from k18hACE2 mice uninfected or infected with 5 × 10^4^ PFU 7 days after administration. Bar is 200 μm. (B) Western blot analysis of TPH2 expression in tissue lysate of the VTA in the brain of k18hACE2 mice uninfected or infected with 5 × 10^4^ PFU 7 days after administration. The intensity of the bands corresponding to TPH2 was normalized to that of β-actin. The data are representative of 6 mice per group, **P < 0.01.

Long-COVID caused by SARS-CoV-2 infection is primarily associated with depression and lethargy, suggesting that neurotransmitters, such as serotonin, are involved. Therefore, we focused on TPH2, the rate-limiting enzyme in serotonin synthesis, which has been reported to be highly active in the VTA. SARS-CoV-2-infected cells of the VTA were examined by fluorescent immunostaining, which revealed TPH2-positive cells (Fig 2 and S1 Fig). To determine the effect of SARS-CoV-2 on TPH2 expression, we analyzed TPH2 expression in the VTA used immunohistochemistry and WB analysis. The results indicated that the VTA of SARS-CoV-2-infected mice exhibited reduced TPH2 expression (Fig 3A and 3B, *P*<0.01).

### SARS-CoV-2 infection induces neuronal cell death in the ventral tegmental area

**Fig 4:**
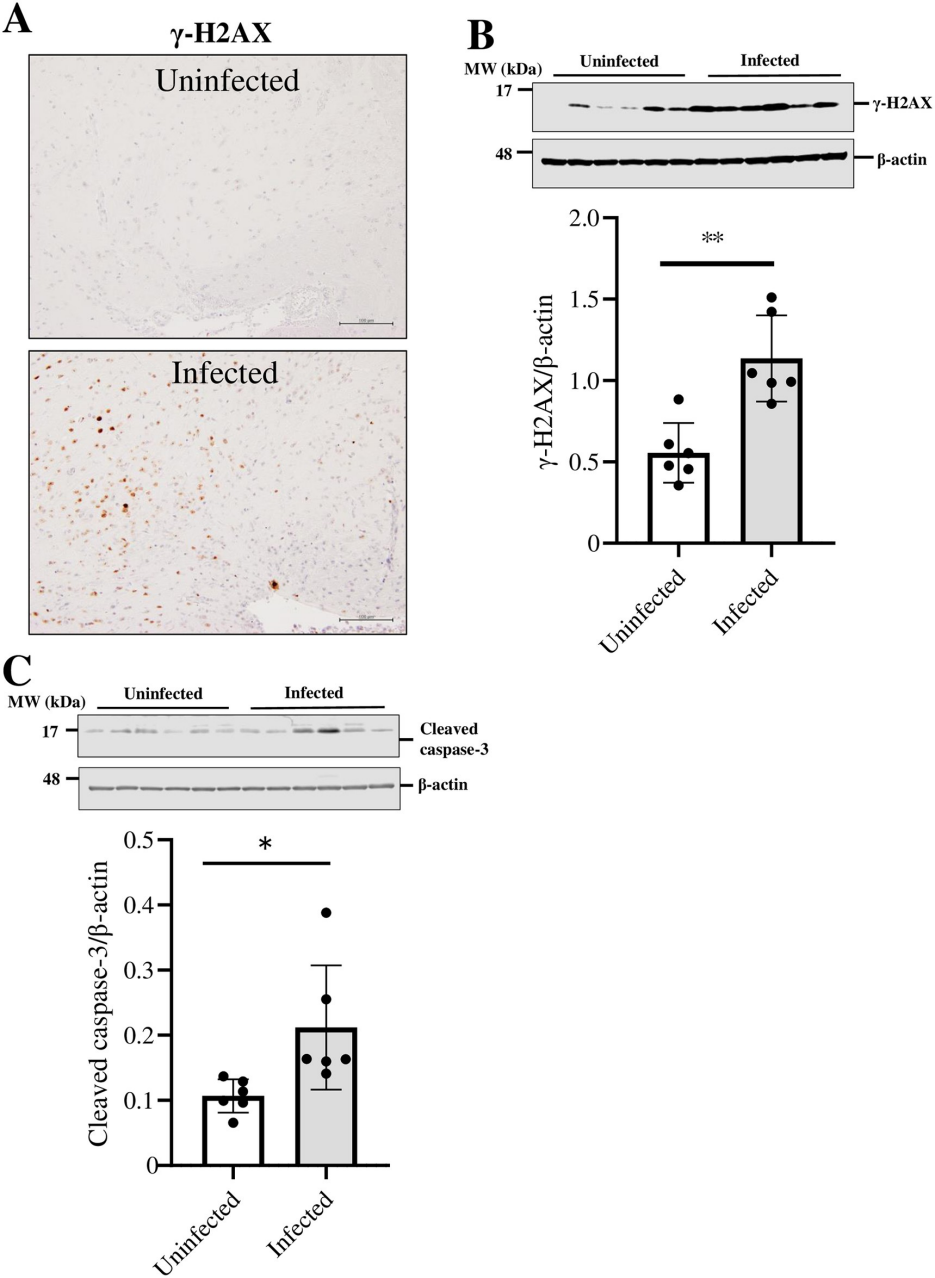
Effect of SARS-CoV-2 infection on neuronal death-related molecules in k18hACE2 mice ventral tegmental area. γ-H2AX in the VTA of brain sections from k18hACE2 mice uninfected or infected with 5 × 10^4^ PFU 6 days after administration. Bar is 100 μm. Western blot analysis of (B) γ-H2AX tissue lysates of the VTA from the brains of k18hACE2 mice uninfected or infected with 5×10^4^ PFU 7 days after administration. The intensity of the bands corresponding to γ-H2AX was normalized to that of β-Actin. The data are representative of 6 mice per group, *P < 0.05, **P < 0.01.

**Fig 5:**
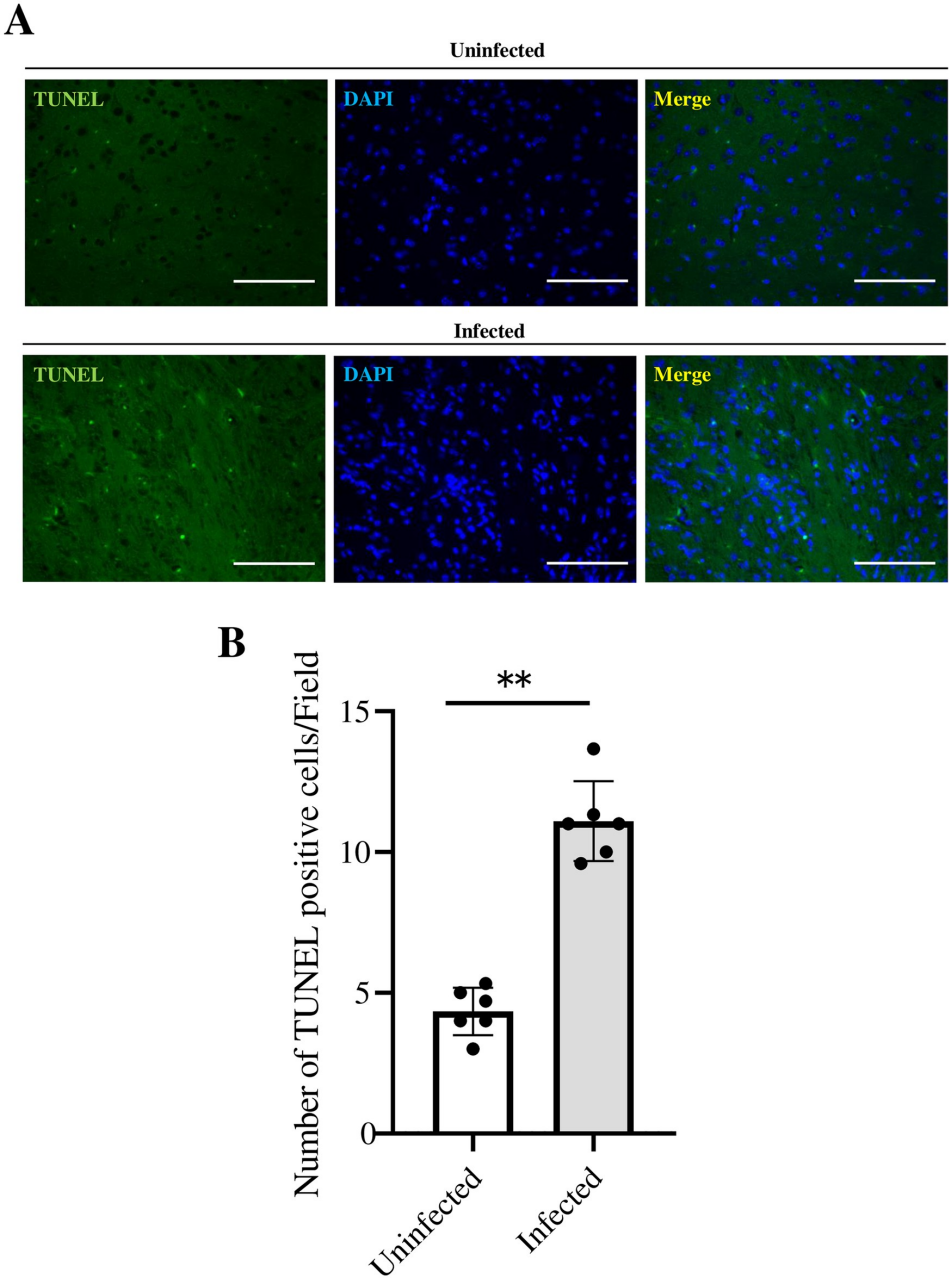
TUNEL staining of SARS-CoV-2 infected k18hACE2 mice ventral tegmental area. (A)TUNEL positive cells in the VTA of brain sections from k18hACE2 mice uninfected or infected with 5×10^4^ PFU 7 days after administration. (B) Fluorescent image taken of three random microscope fields, and the number of TUNEL positive cells were counted in each image. The average of the three fields was then calculated and the result. The data are representative of 6 mice per group, **P<0.01.

Based on these findings, we hypothesized that the infection of TPH2-positive cells with SARS-CoV-2 induces neuronal cell death, thereby causing a decrease in TPH2 expression. γH2AX represents the specific phosphorylation of H2AX, Cleaved caspase-3 and TUNEL positive nuclei in response to DNA damage is considered an indicator of apoptosis and cell death [20, 21]. Therefore, we examined γH2AX, Cleaved caspase-3 and TUNEL positive nuclei as an indicator of neuronal cell death. γH2AX and Cleaved caspase-3 were increased in the VTA of SARS-CoV-2-infected mice compared with the VTA of uninfected mice (Fig 4A–4C, *P*<0.01, *P* = 0.026). And also, TUNEL staining revealed that TUNEL positive nuclei was increased in the VTA of SARS-CoV-2-infected mice (Fig 5A and 5B, S1 File, *P*<0.01) which indicates the induction of neuronal cell death.

### SARS-CoV-2 infection increases phosphorylated tau accumulation through the activation of GSK3β

**Fig 6:**
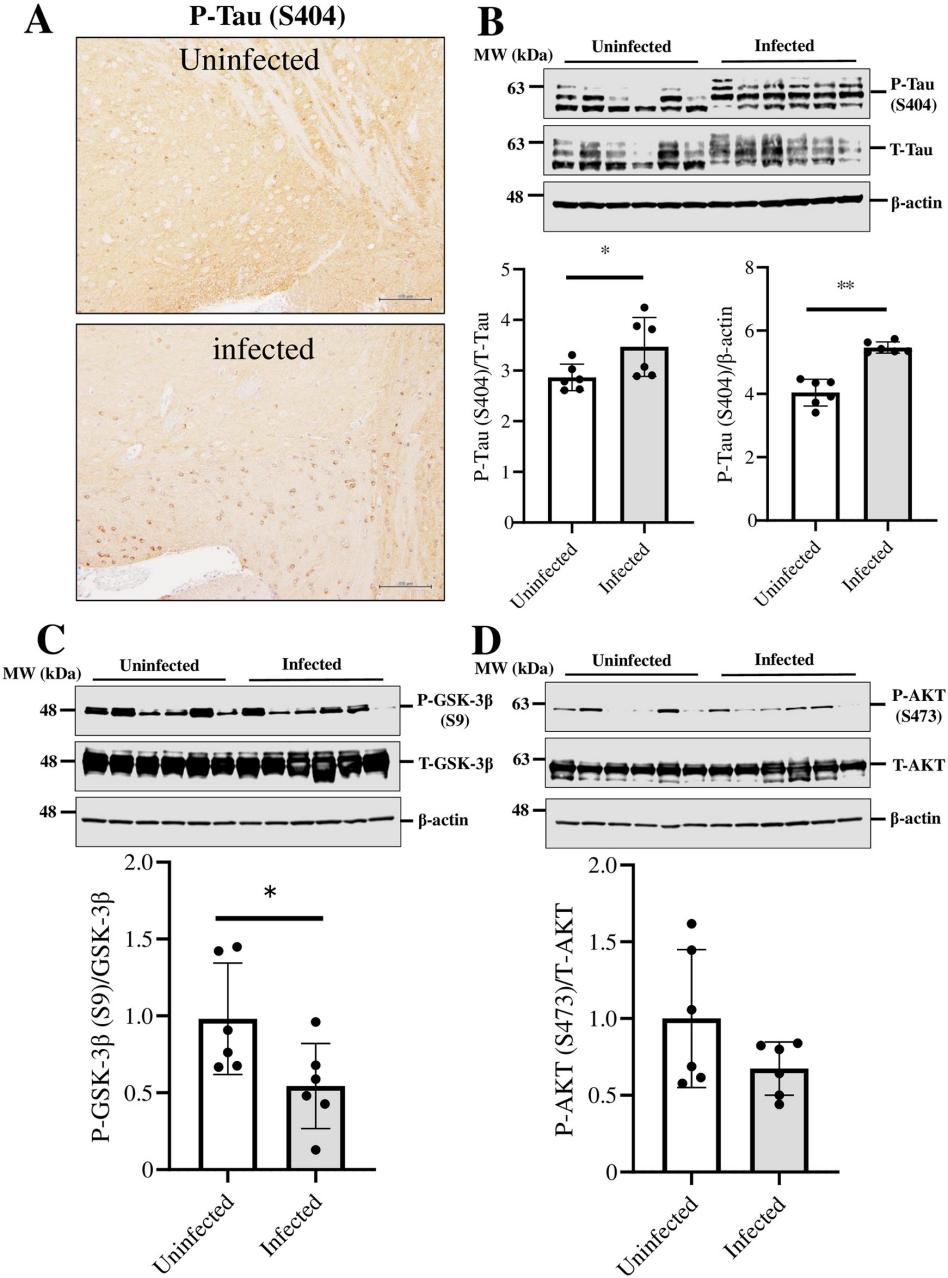
The effect of SARS-CoV-2 infection on neurodegeneration-related molecules in the k18hACE2 mice ventral tegmental area. Immunohistochemical staining analysis of (A) P-Tau (S404) in the VTA of brain sections from k18hACE2 mice uninfected or infected with 5 × 10^4^ PFU 7 days after administration. Bar is 100 μm. Western blot analysis of (B) P-Tau (S404), (B) P-GSK-3β, and (C) P-AKT tissue lysates of the VTA of the brain in k18hACE2 mice uninfected or infected with 5 × 10^4^ PFU 7 Days after administration. The intensity of the bands corresponding to P-Tau (S404), P-GSK-3β, and P-AKT was normalized to that of the corresponding total protein bands. The intensity of the bands corresponding to P-Tau (S404) was normalized to that of β-Actin. The data are representative of 6 mice per group, *P < 0.05, **P < 0.01.

Tau proteins induce neuronal cell death through their excessive phosphorylation and aggregation [21]. We hypothesized that neuronal cell death observed in SARS-CoV-2-infected mice results from the accumulation of phosphorylated tau. Therefore, we examined the phosphorylation and aggregation of tau during SARS-CoV-2 infection to determine the cause of decreased TPH2 expression. Using immunohistochemical staining, we observed that the accumulation of phosphorylated tau was increased in the VTA of SARS-CoV-2 infected mice compared with that of uninfected mice (Fig 6A). Western blot analysis also showed enhanced phosphorylation of tau (*P* = 0.044) and increased expression of phosphorylated tau (*P*<0.01) was observed in the VTA (Fig 6B).

Previous reports have demonstrated that tau phosphorylation and accumulation are regulated by the activation of GSK3β [22]; therefore, we analyzed the phosphorylation of GSK3β at Ser9. Phosphorylation of GSK3β (Ser9) was decreased in the VTA of SARS-CoV-2-infected mice compared with the VTA of uninfected mice (Fig 6C, P = 0.041), indicating that GSK3β is activated during SARS-CoV-2 infection. The phosphorylation of AKT at S478 has been reported to suppress GSK3β activation [23]. We found that the phosphorylation of AKT (Ser473) tended to decrease in the VTA of SARS Cov2-infected mice (Fig 6D).

## Discussion

Over 50% patients infected with SARS-CoV2 display neurological symptoms characterized by headache, dizziness, sleep disturbances, psychosis, anxiety, and depression during recovery [24]. These symptoms are experienced by a significant number of individuals who have recovered from SARS-CoV-2 infection [25]. CNS damage resulting from SARS-CoV-2 infection could be attributed to systemic inflammation [26] or direct infection of neurons [27]; however, the molecular mechanism is still unknown. Therefore, we determined whether intranasal administration of SARS-CoV-2 could infect the CNS of k18hACE2 mice and examined the effect of SARS-CoV-2 infection on the brain nervous system. Our findings indicate that SARS-CoV-2 infected both the olfactory bulb and the cerebral cortex. Unexpectedly, we also observed viral infection in the VTA, a region implicated in neuropsychiatric disorders, including retribution seeking, motivation, and depression [28]. Furthermore, studies in rats indicate that TPH2 activity in the VTA is higher than in other parts of the brain except for the raphe nucleus, a key component in serotonin synthesis [29]. Serotonin is a neurotransmitter integral to mental stability with a strong correlation to condition such as depression, anxiety, and insomnia [7, 30]. However, Carkaci-Salli et al. also reported that TPH2 expression in the VTA is reported to be much lower than in the raphe nucleus, with mRNA expression being 1/10 of that in the raphe nucleus [29]. Therefore, it is possible that serotonin synthesis and TPH2 expression is not observed at sites other than the raphe nucleus. We also need to consider that there are reports of no TPH2 expression in VTA. Nevertheless, under our experimental conditions, expression was also observed in VTA, although it was lower than in Raphe (S2 Fig). A decrease in the expression of TPH2, the rate-limiting enzyme for serotonin production, results in decreased serotonin synthesis, potentially triggering depression and a decline in mental status [31, 32]. In addition, our focus on VTA as previous reports have linked SARS-CoV-2 infection to be involved in the exacerbation and development of depression, psychiatric symptoms, and symptoms of various CNS neurodegenerative diseases [33].

We found that TPH2-positive cells in the VTA also express SARS-CoV-2 spike protein, suggest that TPH2-positive cells are infected with SARS-CoV-2. Furthermore, we found that TPH2 expression was downregulated in the VTA infected with SARS-CoV-2. These results suggest that SARS-CoV-2 infection may decrease serotonin synthesis resulting from decreased TPH2 expression may exacerbate depression and mental status.

Next, we examined the mechanism responsible for the decrease in TPH2 expression during SARS-CoV-2 infection. Previous studies have shown that SARS-CoV-2 infection induces neuronal cell death [34]. Therefore, we analyzed the expression of phosphorylated H2AX (γH2AX), Cleaved caspase-3 and TUNEL positive nuclei to determine whether SARS-CoV-2 infection induces neuronal cell death. Immunohistochemistry and western blot analysis indicated that γH2AX expression was upregulated by SARS-CoV-2 infection. These results suggest that SARS-CoV-2 infection in VTA induces neuronal cell death. The hippocampus is also closely associated with neurological symptoms. However, we found no increase in TUNEL-positive cell nuclei in the hippocampus of SARS-CoV-2-infected mice under our experimental conditions (S3 Fig).

Based on our results, we suggest that SARS-CoV-2 infection causes neuronal death and is involved in the development of neurological symptoms through decreasing TPH2 expression in the VTA. Next, we investigated the mechanism of SARS-CoV-2 infection-induced neuronal death. We focused on the phosphorylation of tau, which is involved in the pathogenesis of CNS neurodegenerative diseases, such as Alzheimer’s disease (AD) and Parkinson’s disease (PD), and analyzed brain tissue from SARS-CoV-2-infected mice by immunohistochemistry and western blotting. The results indicated that phosphorylated tau in the brain tissue is increased by SARS-CoV-2 infection. These results suggest that SARS-CoV-2 infection increases tau phosphorylation, which is associated with neuronal cell death.

Subsequently, we assessed the phosphorylation of GSK3β, one of the major tau kinase, to determine whether its level were elevated due to SARS-CoV-2 infection. We found that SARS-CoV-2 infection decreased the phosphorylation of GSK3β-Ser9. Phosphorylation of the Ser9 site [35] of GSK-3β decreases its kinase activity and is catalyzed primarily by AKT [36]. However, contrary to our expectations, we observed was no significant inhibition of AKT phosphorylation during SARS-CoV-2 infection.

GSK3β plays an important role in tau hyperphosphorylation, synaptic plasticity impairment, and memory impairment [37, 38]. GSK3β activation is induced and tau phosphorylation is enhanced in AD and PD. Furthermore, phosphorylated tau forms aggregate and accumulate intracellularly, inducing apoptosis and neuronal cell death [39, 40]. Thus, our findings suggest that SARS-CoV-2 infection may induce neuronal cell death through the induction of GSK3β-mediated tau phosphorylation in the VTA.

In this study, we focused on TPH2-positive cells expressing spike protein. However, Figs 1 and 2 indicate that there are other cells expressing spike protein besides TPH2-positive cells. Also, infection of microglia has been reported. Therefore, it is necessary in the future to consider the effects of SARS-CoV-2 infection on neuropsychiatric symptoms in addition to the decrease in TPH2 expression, as well as the effects of SARS-CoV-2 infection on various cell types.

Furthermore, we used an acute model of short-term infection in k18hACE2 mice with the WK-521 (wild-type) strain of SARS-CoV-2. Previous reports on the efficiency of infection of k18hACE2 mice with SARS-CoV2 virus strains showed that the wild-type, Delta, and Omicron strains caused lung infection, however, the Omicron strain did not show infection of the brain [41]. In the development of sequelae in human COVID-19 patients, they were also reported to be less infected with the Omicron strain compared to the wild-type and Delta strain [42, 43]. Based on these findings, we speculate that there are strain-specific differences in the SARS-CoV-2 virus-induced brain pathogenesis. Furthermore, Cantuti-Castelvetri *et al*. reported that infects neurons not only via ACE2 but also via neuropilin-1 [44]. In addition, Kong *et al*. reported that SARS-CoV-2 can infect human iPS-derived astrocytes and organoids via neuropilin-1 [6]. Considering these facts, it should be noted that the brain pathogenesis of SARS-CoV-2-infected k18hACE2 mice may differ from clinical symptoms in wild-type mice and humans. Furthermore, k18hACE2 mice model may not be suitable for studying the local brain effects of SARS-CoV2 infection because it expresses ACE2 in ectopic areas of the brain. Nevertheless, the model of infection of k18hACE2 mice with the WK-521 (wild-type) strain of SARS-CoV-2 may provide crucial information on the effects of SARS-CoV-2 on the brain. Moreover, as shown in this study, the experimental findings that SARS-CoV-2 infects neurons in the VTA and induces neuronal cell death may help to elucidate the molecular mechanisms of the abnormalities in the brain nervous system in human COVID-19 patients.

In conclusion, infection of SARS-CoV-2 in TPH2-positive cells of the brain VTA may result in increased phosphorylation of tau through activation of GSK3β, induction of neuronal cell death, suppression of TPH2 expression, and decreased serotonin synthesis. However, measurement of 5-HT and behavioral experiments are needed in the future to further confirm that SARS-CoV-2 infection of VTA causes neurological symptoms via a decrease in TPH2. In addition, the present study shows that SARS-CoV-2 also infects and induced neuronal cell apoptosis in the olfactory bulb and cerebral cortex (S4 Fig, S1 File). AD patients had also been shown to increase β-amyloid and neurotoxicity due to oxidative stress, infection of the brain with SARS-CoV-2 [45]. It is also suggested that infection of the brain with SARS-CoV-2 may be associated with the development of neurological diseases such as AD and PD. Therefore, it is important to analyze both short- and long-term effects of SARS-CoV-2 infection on the brain in the future.

## Supporting information

S1 Fig SARS-CoV-2 infected Tryptophan hydroxylase 2 (TPH2)-positive cells in the ventral tegmental area. Immunohistochemistry staining analysis of SARS-CoV-2 spike protein: red, TPH2: green, and DAPI: blue in the ventral tegmental area of brain sections from k18hACE2 mice uninfected or infected with 5×10^4^ PFU 7 days after administration. Bar is 50 μm.
(TIF)

S2 Fig Comparison of TPH2 expression in the ventral tegmental area and raphe. Immunohistochemistry staining analysis of TPH2 in the (A) Ventral tegmental area and (B) Raphe of the brain section from SARS-CoV-2 uninfected k18hACE2 mice. Bar is 50 μm.
(TIF)

S3 Fig TUNEL staining of SARS-CoV-2 infected k18hACE2 mice hippocampus. TUNEL positive cells in hippocamps of brain sections from k18hACE2-mice uninfected or infected with 5×10^4^ PFU 7 days after administration. Bar is 100 μm.
(TIF)

S4 Fig TUNEL staining of SARS-CoV-2 infected k18hACE2 mice cerebral cortex and olfactory blub. TUNEL positive cells in (A) cerebral cortex and (C) olfactory blub of brain sections from k18hACE2-mice uninfected or infected with 5×10^4^ PFU 7 days after administration. Fluorescent image taken of three random microscope fields, and the number of TUNEL positive nuclei were counted in each image. The average of the three fields was then calculated and the result of (B) cerebral cortex and (D). The data are representative of 6 mice per group, **P<0.01.
(TIF)

S1 Raw images (PDF)

S1 File Intensity of western blotting bands and counting of TUNEL positive cells. (XLSX)

## References

[1] Patel UK, Mehta N, Patel A, Patel N, Ortiz JF, Khurana M, et al. Long-Term Neurological Sequelae Among Severe COVID-19 Patients: A Systematic Review and Meta-Analysis. Cureus. 2022 Sep 28;14(9):e29694. doi: 10.7759/cureus.29694 36321004 PMC9616013

[2] Zawilska JB, Kuczyńska K. Psychiatric and neurological complications of long COVID. J Psychiatr Res. 2022 Dec;156:349–360. Epub 2022 Oct 20. doi: 10.1016/j.jpsychires.2022.10.045 36326545 PMC9582925

[3] Elizalde-Díaz JP, Miranda-Narváez CL, Martínez-Lazcano JC, Martínez-Martínez E. The relationship between chronic immune response and neurodegenerative damage in long COVID-19. Front Immunol. 2022 Dec 16;13:1039427. doi: 10.3389/fimmu.2022.1039427 36591299 PMC9800881

[4] Crunfli F, Carregari VC, Veras FP, Silva LS, Nogueira MH, Antunes ASLM, et al. Morphological, cellular, and molecular basis of brain infection in COVID-19 patients. Proc Natl Acad Sci U S A. 2022 Aug 30;119(35):e2200960119. Epub 2022 Aug 11. doi: 10.1073/pnas.2200960119 35951647 PMC9436354

[5] Savelieff MG, Feldman EL, Stino AM. Neurological sequela and disruption of neuron-glia homeostasis in SARS-CoV-2 infection. Neurobiol Dis. 2022 Jun 15;168:105715. Epub 2022 Mar 29. doi: 10.1016/j.nbd.2022.105715 35364273 PMC8963977

[6] Kong W, Montano M, Corley MJ, Helmy E, Kobayashi H, Kinisu M, et al. Neuropilin-1 mediates SARS-CoV-2 infection of astrocytes in brain organoids, inducing inflammation leading to dysfunction and death of neurons. mBio. 2022 Dec 20;13(6):e0230822. Epub 2022 Oct 31. doi: 10.1128/mbio.02308-22 36314791 PMC9765283

[7] Kraus C, Castrén E, Kasper S, Lanzenberger R. Serotonin and neuroplasticity—Links between molecular, functional and structural pathophysiology in depression. Neurosci Biobehav Rev. 2017 Jun;77:317–326. Epub 2017 Mar 22. doi: 10.1016/j.neubiorev.2017.03.007 28342763

[8] Chakraborty S, Lennon JC, Malkaram SA, Zeng Y, Fisher DW, Dong H. Serotonergic system, cognition, and BPSD in Alzheimer’s disease. Neurosci Lett. 2019 Jun 21;704:36–44. Epub 2019 Apr 1. doi: 10.1016/j.neulet.2019.03.050 30946928 PMC6594906

[9] Sadlier C, Albrich WC, Neogi U, Lunjani N, Horgan M, O’Toole PW, et al. Metabolic rewiring and serotonin depletion in patients with postacute sequelae of COVID-19. Allergy. 2022 May;77(5):1623–1625. Epub 2022 Feb 17. doi: 10.1111/all.15253 35150456 PMC9111264

[10] Sen A. Does serotonin deficiency lead to anosmia, ageusia, dysfunctional chemesthesis and increased severity of illness in COVID-19? Med Hypotheses. 2021 Aug;153:110627. Epub 2021 Jun 6. doi: 10.1016/j.mehy.2021.110627 34139598 PMC8180092

[11] Gutknecht L, Popp S, Waider J, Sommerlandt FM, Göppner C, Post A, et al. Interaction of brain 5-HT synthesis deficiency, chronic stress and sex differentially impact emotional behavior in Tph2 knockout mice. Psychopharmacology (Berl). 2015 Jul;232(14):2429–41. Epub 2015 Feb 27. doi: 10.1007/s00213-015-3879-0 25716307 PMC4480945

[12] Premraj L, Kannapadi NV, Briggs J, Seal SM, Battaglini D, Fanning J, et al. Mid and long-term neurological and neuropsychiatric manifestations of post-COVID-19 syndrome: a meta-analysis. J Neurol Sci. 2022 Mar 15;434:120162. Epub 2022 Jan 29. doi: 10.1016/j.jns.2022.120162 35121209 PMC8798975

[13] Ziauddeen N, Gurdasani D, O’Hara ME, Hastie C, Roderick P, Yao G, et al. Characteristics and impact of long covid: findings from an online survey. PLoS One. 2022 Mar 8;17(3):e0264331. doi: 10.1371/journal.pone.0264331 35259179 PMC8903286

[14] Giurgi-Oncu C, Tudoran C, Pop GN, Bredicean C, Pescariu SA, Giurgiuca A, et al. Cardiovascular abnormalities and mental health difficulties result in a reduced quality of life in the post-acute COVID-19 syndrome. Brain Sci. 2021 Nov 2;11(11):1456. doi: 10.3390/brainsci11111456 34827455 PMC8615893

[15] Cheallaigh C, Nadarajan P, McLaughlin AM, Bourke NM, Bergin C, et al. Persistent fatigue following SARS-CoV-2 infection is common and independent of severity of initial infection. PLoS One. 2020 Nov 9;15(11):e0240784. doi: 10.1371/journal.pone.0240784 33166287 PMC7652254

[16] Hassler L, Wysocki J, Ahrendsen JT, Ye M, Gelarden I, Nicolaescu V, et al. Intranasal soluble ACE2 improves survival and prevents brain SARS-CoV-2 infection. Life Sci Alliance. 2023 Apr 11;6(7):e202301969. doi: 10.26508/lsa.202301969 37041017 PMC10098141

[17] Matsuyama S, Nao N, Shirato K, Kawase M, Saito S, Takayama I, et al. Enhanced isolation of SARS-CoV-2 by TMPRSS2-expressing cells. Proc Natl Acad Sci U S A. 2020 Mar 31;117(13):7001–7003. Epub 2020 Mar 12. doi: 10.1073/pnas.2002589117 32165541 PMC7132130

[18] McCray PB Jr, Pewe L, Wohlford-Lenane C, Hickey M, Manzel L, Shi L, et al. Lethal infection of K18-hACE2 mice infected with severe acute respiratory syndrome coronavirus. J Virol. 2007 Jan;81(2):813–21. Epub 2006 Nov 1. doi: 10.1128/JVI.02012-06 17079315 PMC1797474

[19] Imai M, Kawakami F, Kubo M, Kanzaki M, Maruyama H, Kawashima R, et al. LRRK2 Inhibition Ameliorates Dexamethasone-Induced Glucose Intolerance via Prevents Impairment in GLUT4 Membrane Translocation in Adipocytes. Biol Pharm Bull. 2020;43(11):1660–1668. doi: 10.1248/bpb.b20-00377 . 33132310

[20] Sharma A, Singh K, Almasan A. Histone H2AX phosphorylation: a marker for DNA damage. Methods Mol Biol. 2012;920:613–26. doi: 10.1007/978-1-61779-998-3_40 22941631

[21] Chi H, Chang HY, Sang TK. Neuronal cell death mechanisms in major neurodegenerative diseases. J Mol Sci. 2018 Oct 9;19(10):3082. doi: 10.3390/ijms19103082 30304824 PMC6213751

[22] Wisessaowapak C, Visitnonthachai D, Watcharasit P, Satayavivad J. Prolonged arsenic exposure increases tau phosphorylation in differentiated SH-SY5Y cells: the contribution of GSK3 and ERK1/2. Environ Toxicol Pharmacol. 2021 May;84:103626. Epub 2021 Feb 20. doi: 10.1016/j.etap.2021.103626 33621689

[23] Liao S, Wu J, Liu R, Wang S, Luo J, Yang Y, et al. A novel compound DBZ ameliorates neuroinflammation in LPS-stimulated microglia and ischemic stroke rats: role of Akt(Ser473)/GSK3β(Ser9)-mediated Nrf2 activation. Redox Biol. 2020 Sep;36:101644. Epub 2020 Jul 17. doi: 10.1016/j.redox.2020.101644 32863210 PMC7371982

[24] COVID-19 Mental Disorders Collaborators. Global prevalence and burden of depressive and anxiety disorders in 204 countries and territories in 2020 due to the COVID-19 pandemic. Lancet. 2021 Nov 6;398(10312):1700–1712. Epub 2021 Oct 8. doi: 10.1016/S0140-6736(21)02143-7 34634250 PMC8500697

[25] Castanares-Zapatero D, Chalon P, Kohn L, Dauvrin M, Detollenaere J, Maertens de Noordhout C, et al. Pathophysiology and mechanism of long COVID: a comprehensive review. Ann Med. 2022 Dec;54(1):1473–1487. doi: 10.1080/07853890.2022.2076901 35594336 PMC9132392

[26] Hirzel C, Grandgirard D, Surial B, Wider MF, Leppert D, Kuhle J, et al. Neuro-axonal injury in COVID-19: the role of systemic inflammation and SARS-CoV-2 specific immune response. Ther Adv Neurol Disord. 2022 Mar 12;15:17562864221080528. doi: 10.1177/17562864221080528 35299779 PMC8922213

[27] Guo YR, Cao QD, Hong ZS, Tan YY, Chen SD, Jin HJ, et al. The origin, transmission and clinical therapies on coronavirus disease 2019 (COVID-19) outbreak—an update on the status. Mil Med Res. 2020 Mar 13;7(1):11. doi: 10.1186/s40779-020-00240-0 32169119 PMC7068984

[28] Barker DJ, Root DH, Zhang S, Morales M. Multiplexed neurochemical signaling by neurons of the ventral tegmental area. J Chem Neuroanat. 2016 Apr;73:33–42. Epub 2016 Jan 4. doi: 10.1016/j.jchemneu.2015.12.016 26763116 PMC4818729

[29] Carkaci-Salli N, Salli U, Kuntz-Melcavage KL, Pennock MM, Ozgen H, Tekin I, et al. TPH2 in the ventral tegmental area of the male rat brain. Brain Res Bull. 2011 Apr 5;84(6):376–80. Epub 2011 Jan 25. doi: 10.1016/j.brainresbull.2011.01.006 21272616 PMC3070366

[30] Zangrossi H Jr, Graeff FG. Serotonin in anxiety and panic: contributions of the elevated T-maze. Neurosci Biobehav Rev. 2014 Oct;46 Pt 3:397–406. Epub 2014 Mar 21. doi: 10.1016/j.neubiorev.2014.03.007 24657635

[31] Wang B, Shi H, Ren L, Miao Z, Wan B, Yang H, et al. Ahi1 regulates serotonin production by the GR/ERβ/TPH2 pathway involving sexual differences in depressive behaviors. Cell Commun Signal. 2022 May 28;20(1):74. doi: 10.1186/s12964-022-00894-4 35643536 PMC9148486

[32] Zhang X, Beaulieu JM, Sotnikova TD, Gainetdinov RR, Caron MG. Tryptophan hydroxylase-2 controls brain serotonin synthesis. Science. 2004 Jul 9;305(5681):217. doi: 10.1126/science.1097540 15247473

[33] Krey L, Huber MK, Höglinger GU, Wegner F. Can SARS-CoV-2 infection lead to neurodegeneration and Parkinson’s disease? Brain Sci. 2021 Dec 18;11(12):1654. doi: 10.3390/brainsci11121654 34942956 PMC8699589

[34] Mesci P, de Souza JS, Martin-Sancho L, Macia A, Saleh A, Yin X, et al. SARS-CoV-2 infects human brain organoids causing cell death and loss of synapses that can be rescued by treatment with Sofosbuvir. PLoS Biol. 2022 Nov 3;20(11):e3001845. doi: 10.1371/journal.pbio.3001845 36327326 PMC9632769

[35] Frame S, Cohen P, Biondi RM. A common phosphate binding site explains the unique substrate specificity of GSK3 and its inactivation by phosphorylation. Mol Cell. 2001 Jun;7(6):1321–7. doi: 10.1016/s1097-2765(01)00253-2 11430833

[36] Yang W, Liu Y, Xu QQ, Xian YF, Lin ZX. Sulforaphene ameliorates neuroinflammation and hyperphosphorylated Tau protein via regulating the PI3K/Akt/GSK-3β pathway in experimental models of Alzheimer’s disease. Oxid Med Cell Longev. 2020 Sep 10;2020:4754195. doi: 10.1155/2020/4754195 32963694 PMC7502131

[37] Hernandez F, Lucas JJ, Avila J. GSK3 and tau: two convergence points in Alzheimer’s disease. J Alzheimers Dis. 2013;33 Suppl 1:S141–4. doi: 10.3233/JAD-2012-129025 22710914

[38] Medina M, Avila J. Understanding the relationship between GSK-3 and Alzheimer’s disease: a focus on how GSK-3 can modulate synaptic plasticity processes. Expert Rev Neurother. 2013 May;13(5):495–503. doi: 10.1586/ern.13.39 23621307

[39] Buée L, Troquier L, Burnouf S, Belarbi K, Van der Jeugd A, Ahmed T, et al. From tau phosphorylation to tau aggregation: what about neuronal death? Biochem Soc Trans. 2010 Aug;38(4):967–72. doi: 10.1042/BST0380967 20658986

[40] Ma Y, Wang J, Xu D, Chen Y, Han X. Chronic MC-LR exposure promoted Aβ and p-tau accumulation via regulating Akt/GSK-3β signal pathway. Sci Total Environ. 2021 Nov 10;794:148732. Epub 2021 Jun 29. doi: 10.1016/j.scitotenv.2021.148732 34323745

[41] Seehusen F, Clark JJ, Sharma P, Bentley EG, Kirby A, Subramaniam K, et al. Neuroinvasion and neurotropism by SARS-CoV-2 variants in the K18-hACE2 mouse. Viruses. 2022 May 11;14(5):1020. doi: 10.3390/v14051020 35632761 PMC9146514

[42] Antonelli M, Pujol JC, Spector TD, Ourselin S, Steves CJ. Risk of long COVID associated with delta versus omicron variants of SARS-CoV-2. Lancet. 2022 Jun 18;399(10343):2263–2264. doi: 10.1016/S0140-6736(22)00941-2 35717982 PMC9212672

[43] Magnusson K, Kristoffersen DT, Dell’Isola A, Kiadaliri A, Turkiewicz A, Runhaar J, et al. Post-covid medical complaints following infection with SARS-CoV-2 omicron vs delta variants. Nat Commun. 2022 Nov 30;13(1):7363. doi: 10.1038/s41467-022-35240-2 36450749 PMC9709355

[44] Cantuti-Castelvetri L, Ojha R, Pedro LD, Djannatian M, Franz J, Kuivanen S, et al. Neuropilin-1 facilitates SARS-CoV-2 cell entry and infectivity. Science. 2020 Nov 13;370(6518):856–860. Epub 2020 Oct 20. doi: 10.1126/science.abd2985 33082293 PMC7857391

[45] Chiricosta L, Gugliandolo A, Mazzon E. SARS-CoV-2 exacerbates beta-amyloid neurotoxicity, inflammation and oxidative stress in Alzheimer’s disease patients. Int J Mol Sci. 2021 Dec 19;22(24):13603. doi: 10.3390/ijms222413603 . 34948400 PMC8705864

