# Supplementary figures and images for: SARS-CoV-2 propagation to the TPH2-positive neurons in the ventral tegmental area induces cell death via GSK3β-dependent accumulation of phosphorylated tau
Source: PLoS One. 2024 Oct 30;19(10):e0312834. doi: 10.1371/journal.pone.0312834 (PMC11524480; doi:10.1371/journal.pone.0312834)

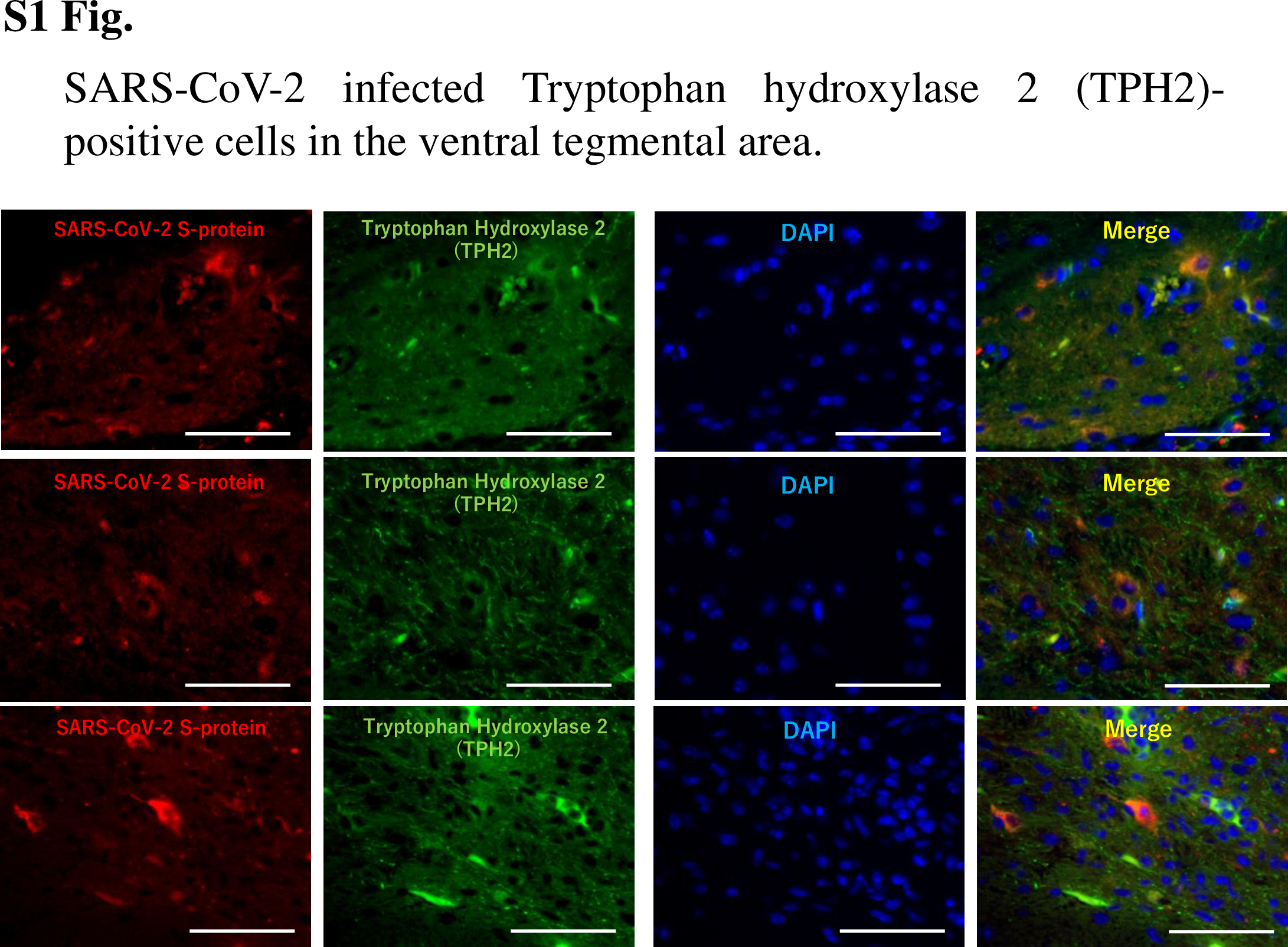

Supplement: S1 Fig — Immunohistochemistry staining analysis of SARS-CoV-2 spike protein: red, TPH2: green, and DAPI: blue in the ventral tegmental area of brain sections from k18hACE2 mice uninfected or infected with 5×10^4^ PFU 7 days after administration. Bar is 50 μm. (TIF) [file pone.0312834.s001.tif]

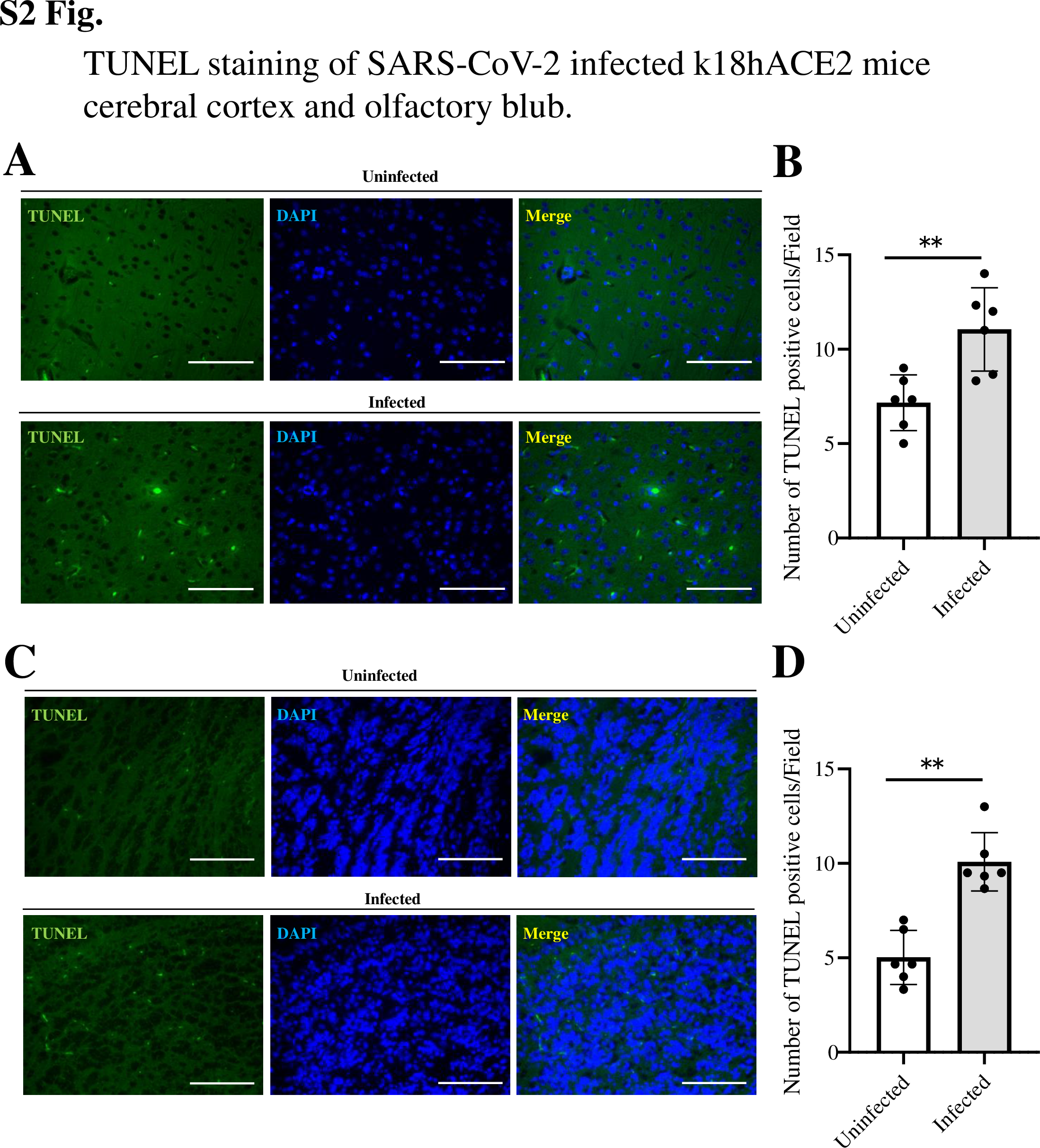

Supplement: S2 Fig — Immunohistochemistry staining analysis of TPH2 in the (A) Ventral tegmental area and (B) Raphe of the brain section from SARS-CoV-2 uninfected k18hACE2 mice. Bar is 50 μm. (TIF) [file pone.0312834.s002.tif]

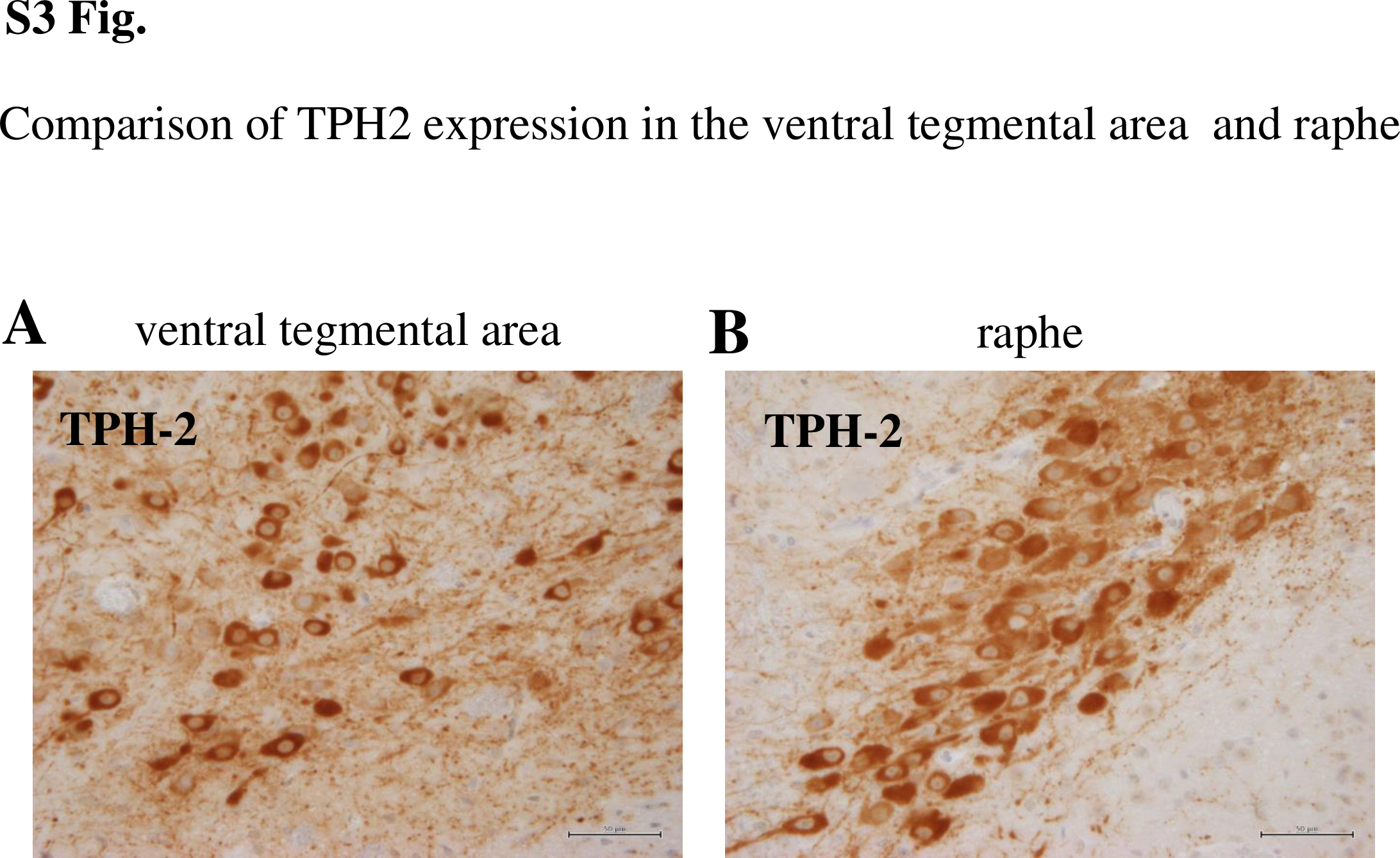

Supplement: S3 Fig — TUNEL positive cells in hippocamps of brain sections from k18hACE2-mice uninfected or infected with 5×10^4^ PFU 7 days after administration. Bar is 100 μm. (TIF) [file pone.0312834.s003.tif]

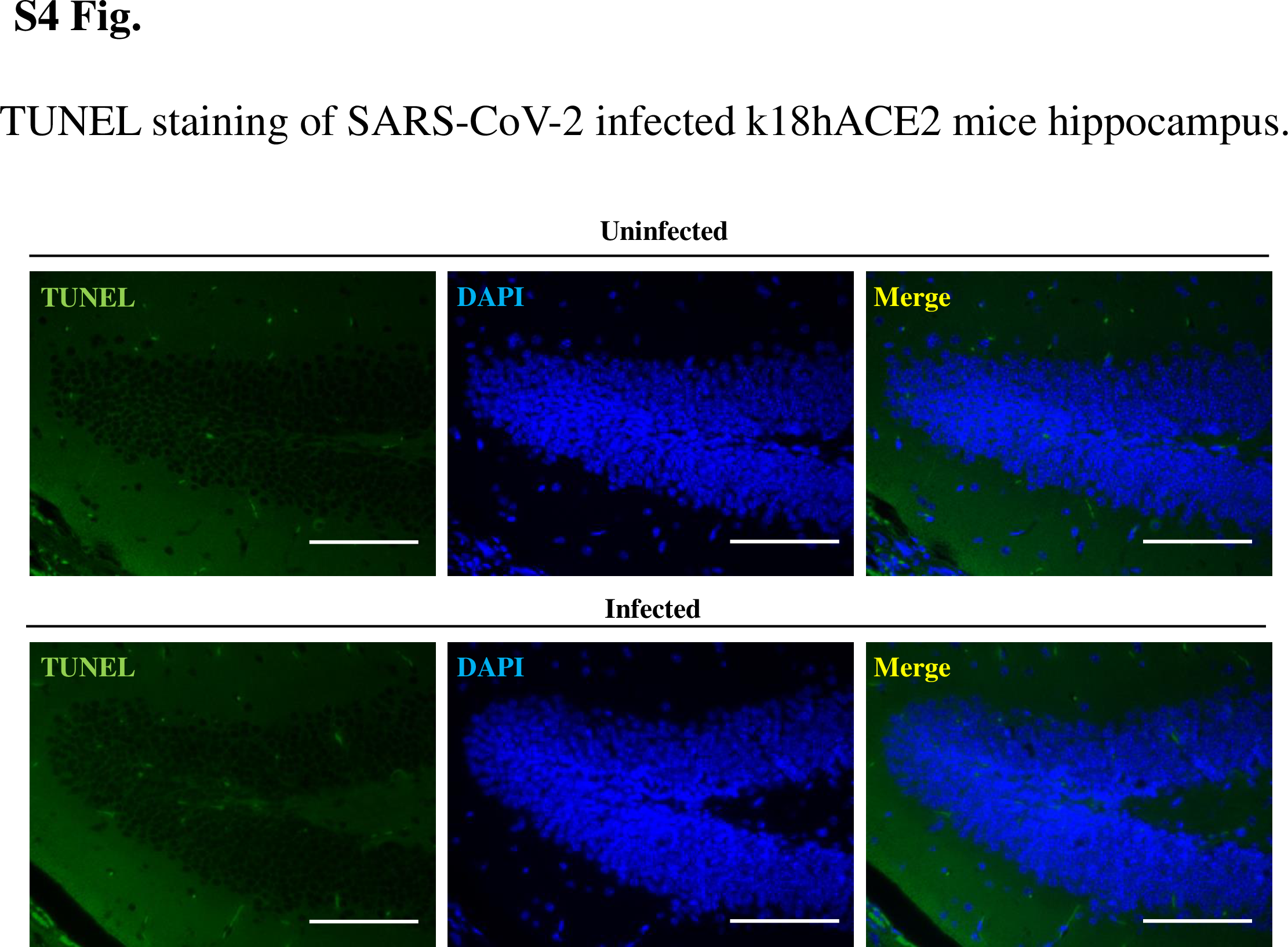

Supplement: S4 Fig — TUNEL positive cells in (A) cerebral cortex and (C) olfactory blub of brain sections from k18hACE2-mice uninfected or infected with 5×10^4^ PFU 7 days after administration. Fluorescent image taken of three random microscope fields, and the number of TUNEL positive nuclei were counted in each image. The average of the three fields was then calculated and the result of (B) cerebral cortex and (D). The data are representative of 6 mice per group, **P<0.01. (TIF) [file pone.0312834.s004.tif]

Fig.1C

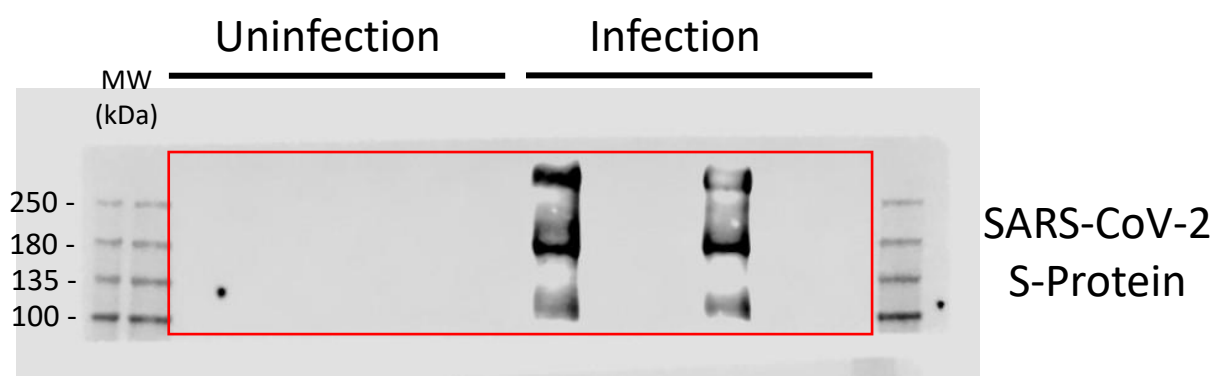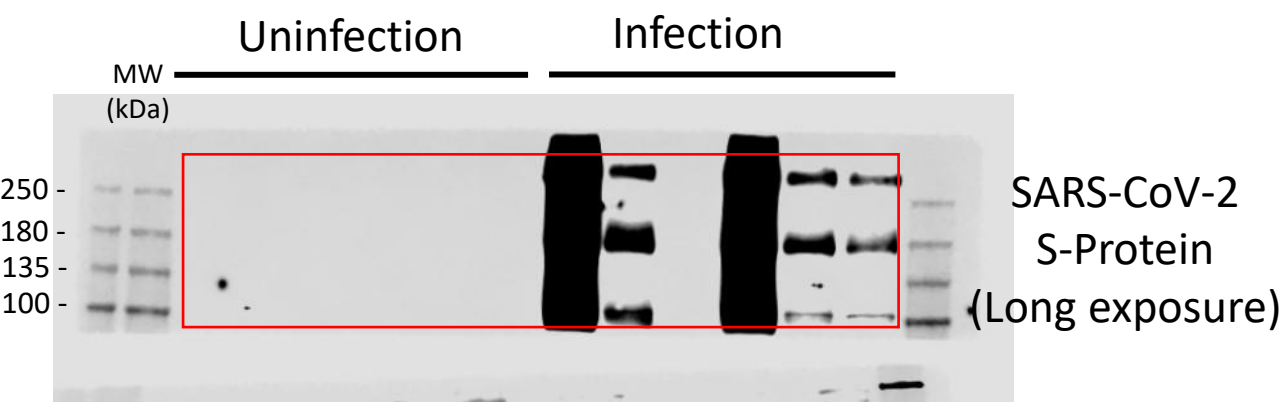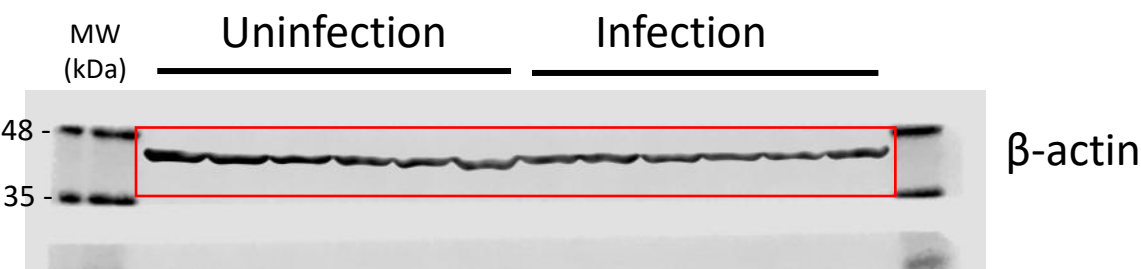

Fig.1D

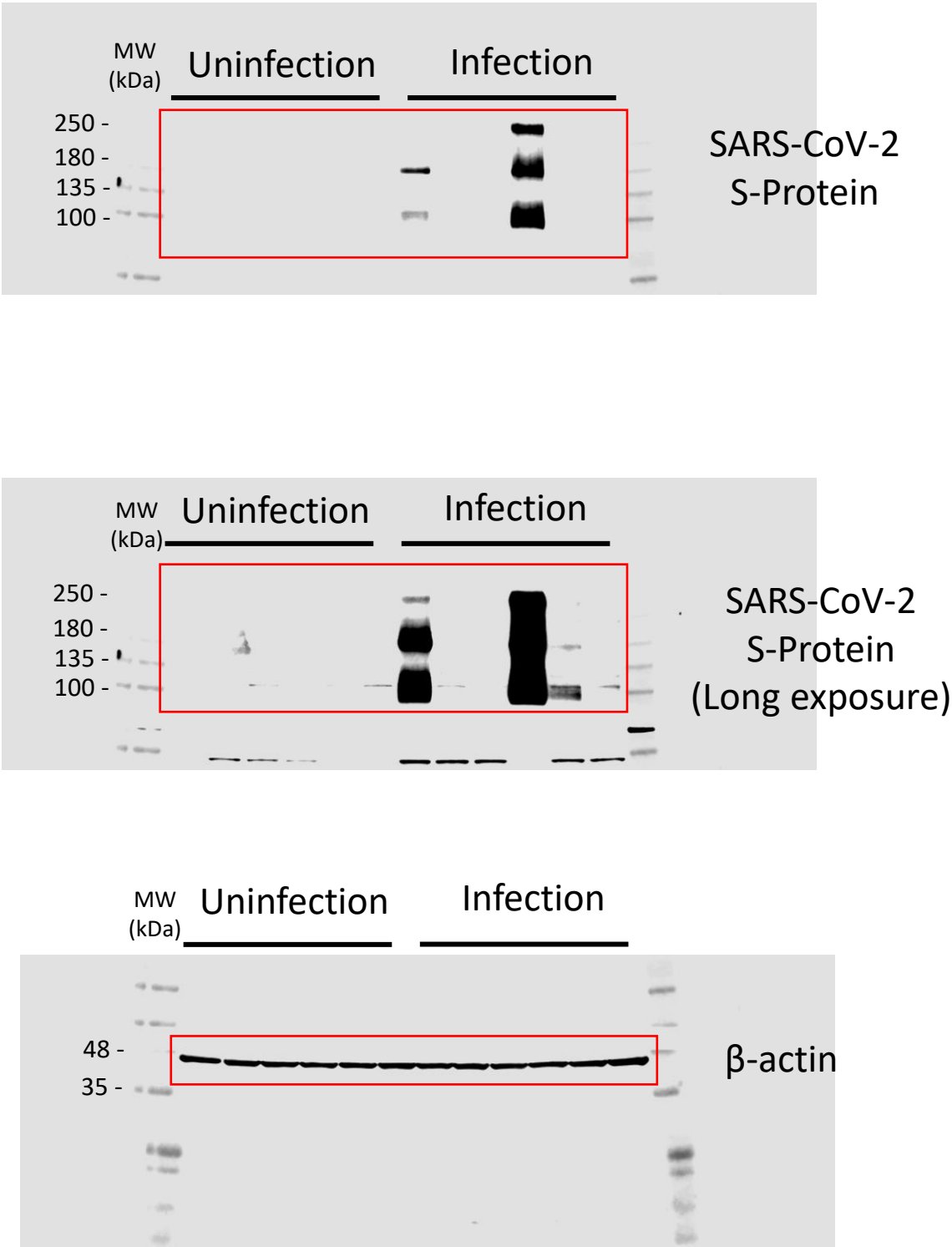

Fig.1E

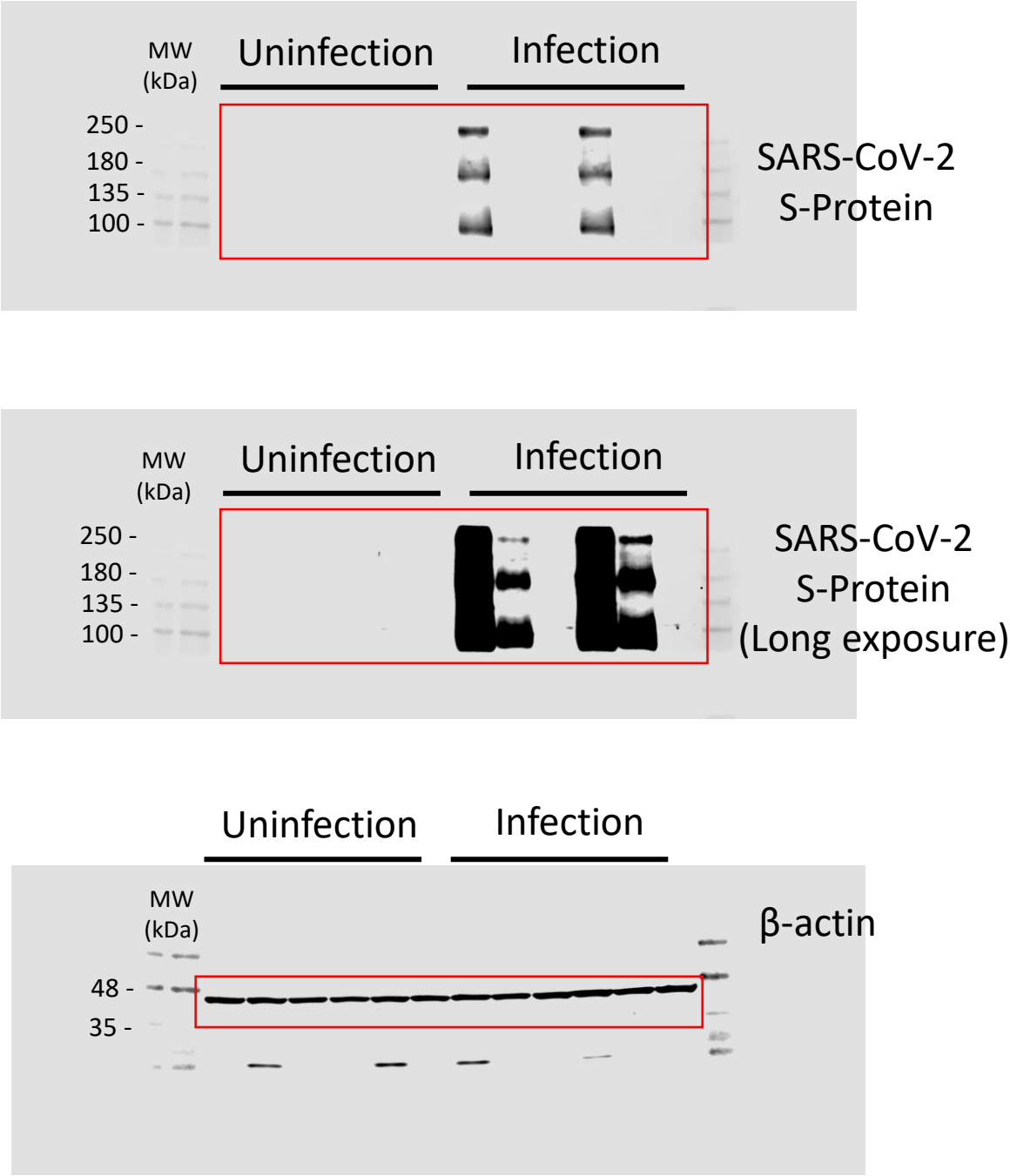

Fig.3B

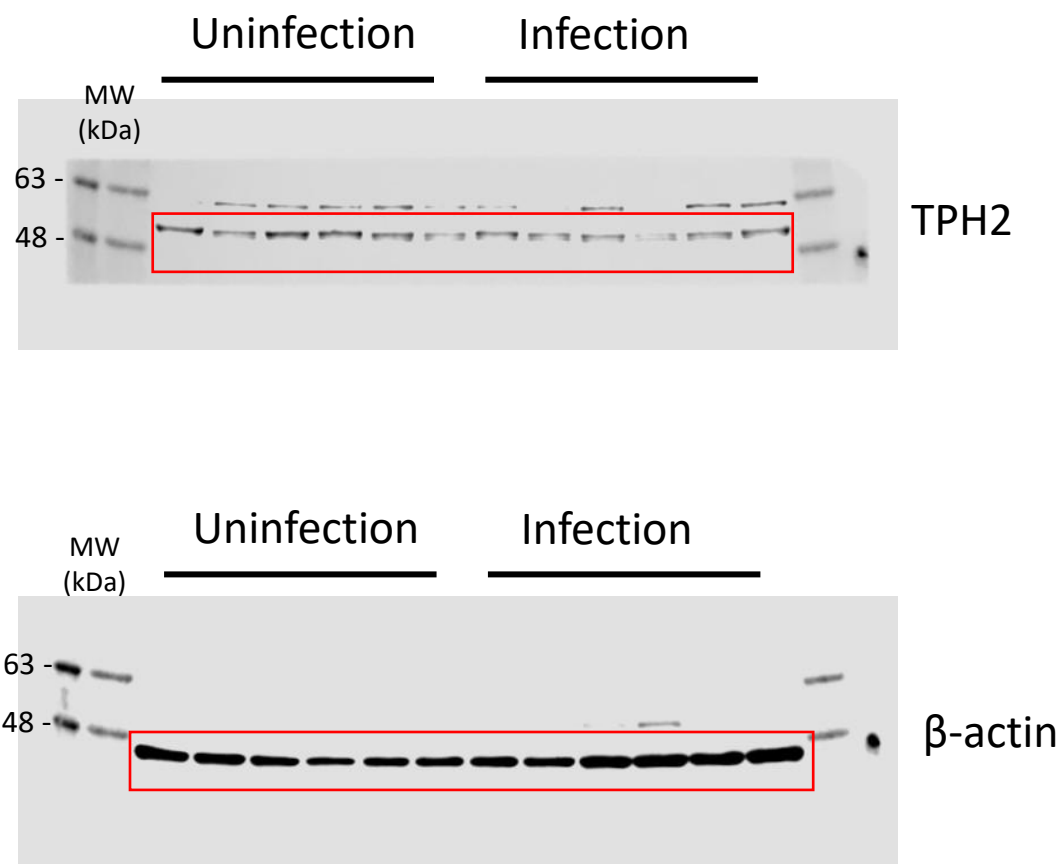

Fig.4B

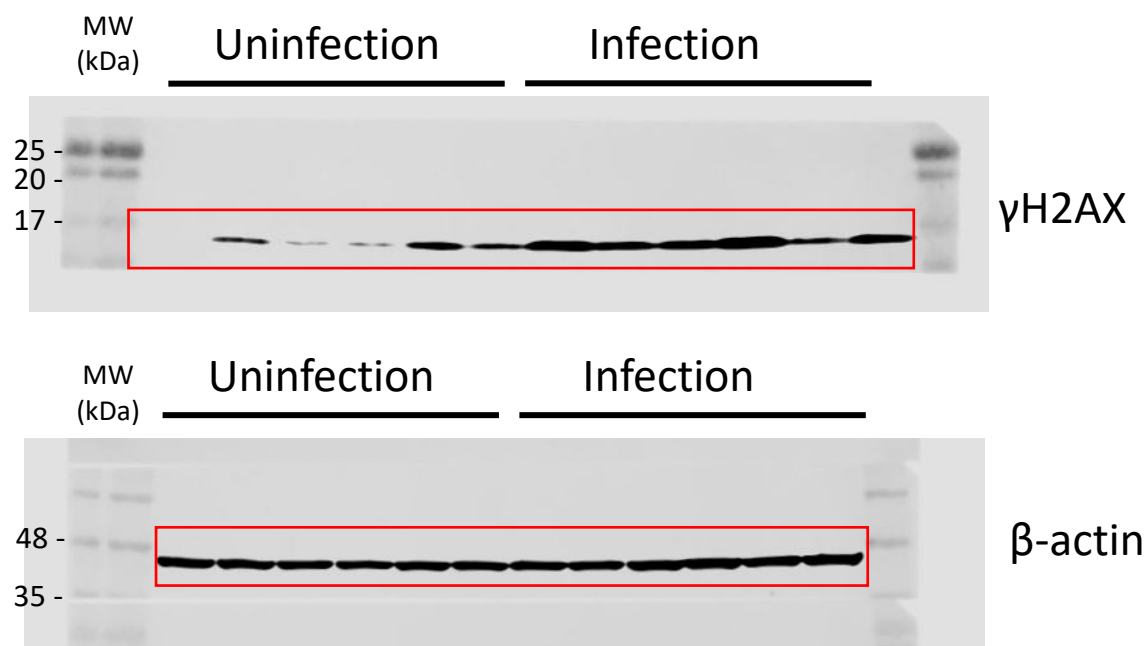

Fig.4C

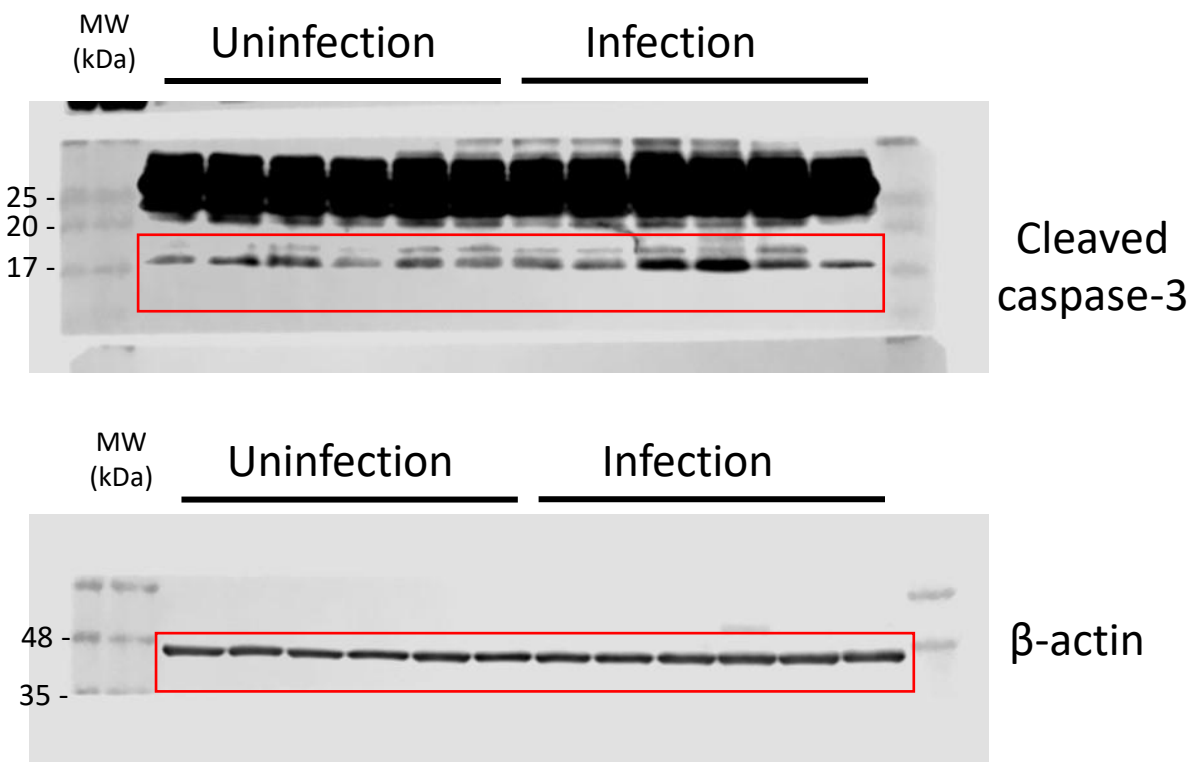

Fig.6B

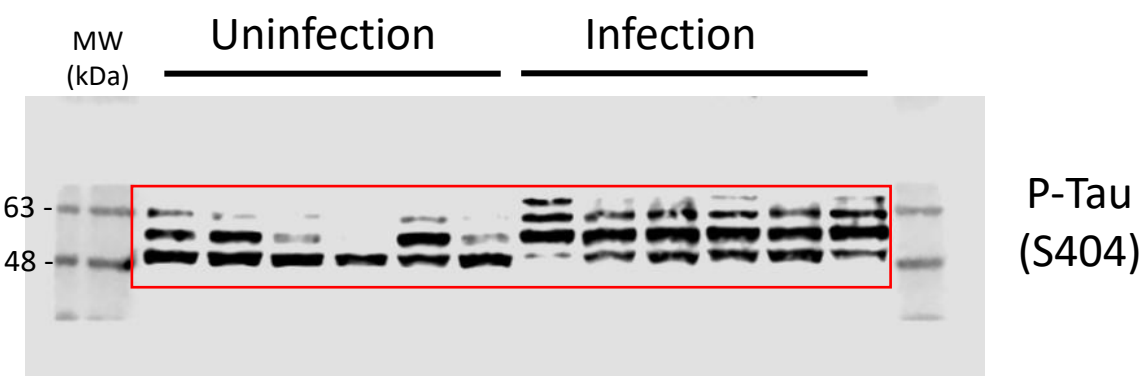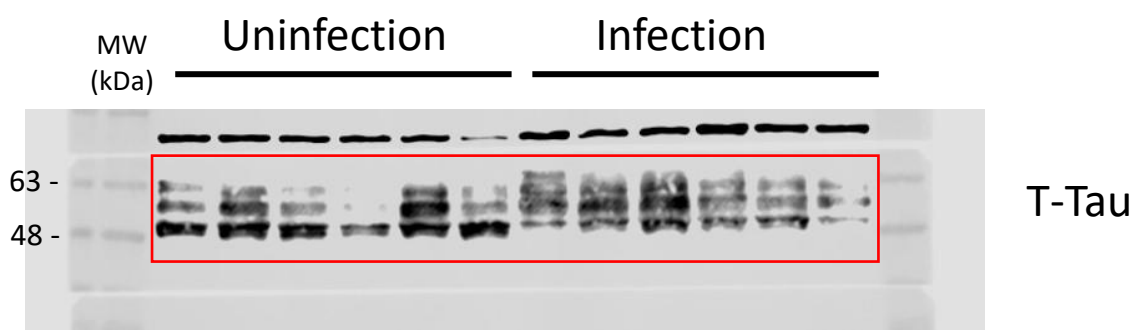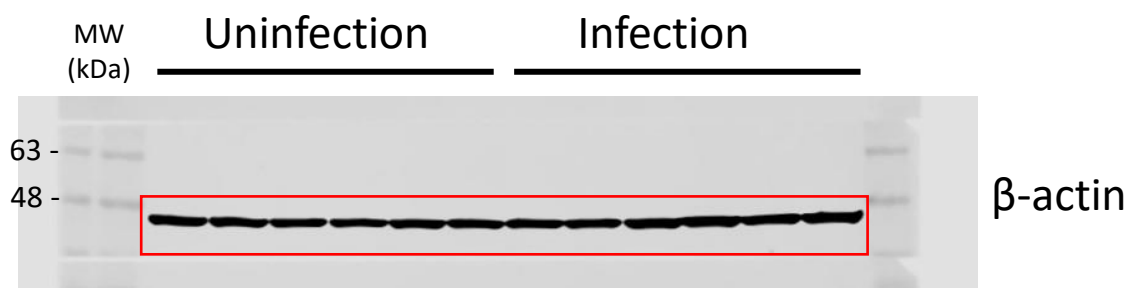

Fig.6C

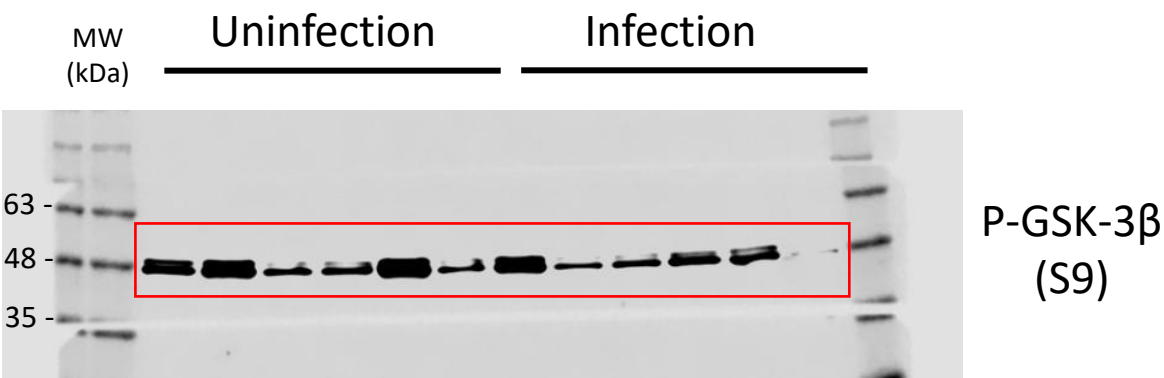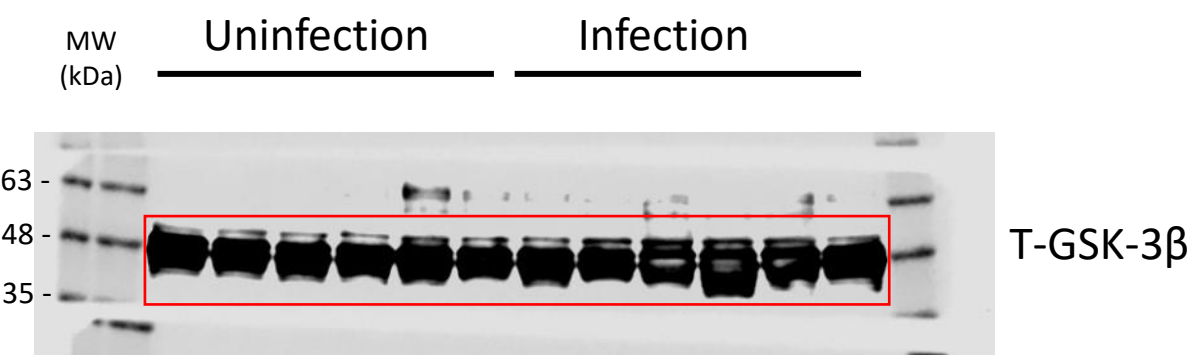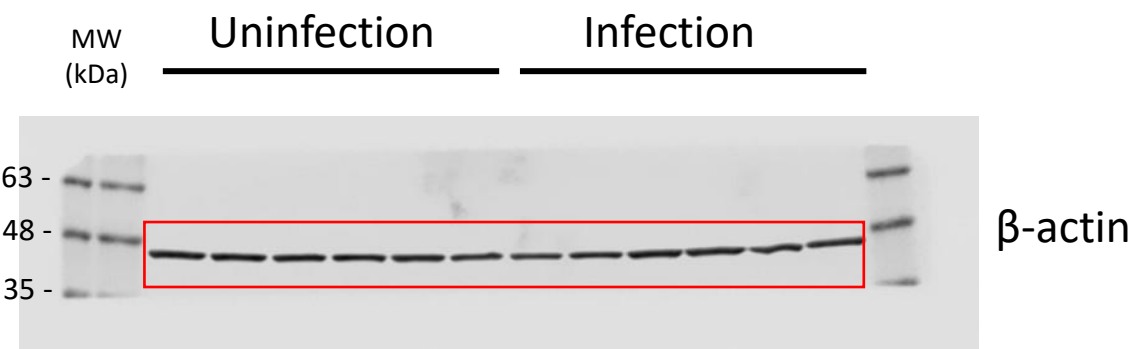

Fig.6D

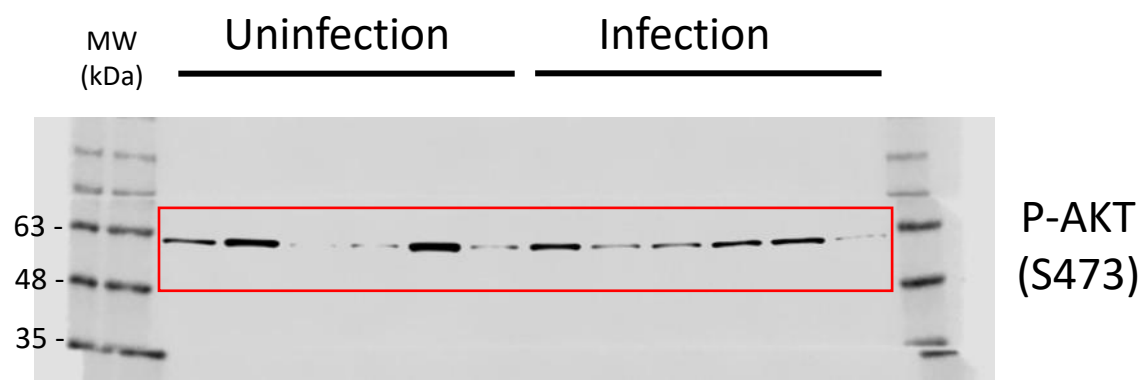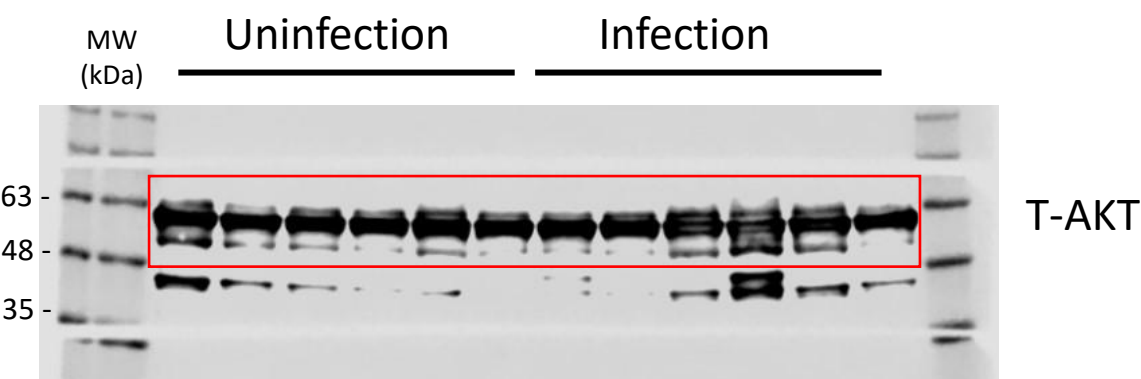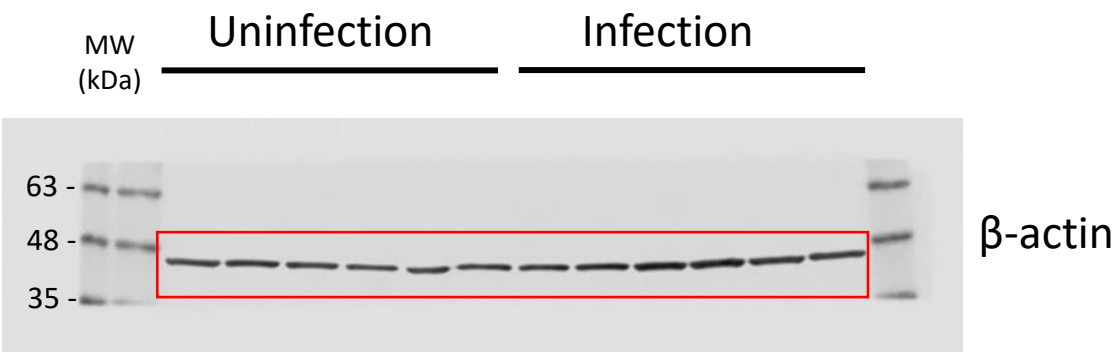

Supplement: S1 Raw images — (PDF) [file pone.0312834.s005.pdf]
